# Global, regional, and national burdens of pertussis among adults: a systematic analysis of age-specific trends using Global Burden of Diseases 2021 data

**DOI:** 10.1186/s40249-025-01355-z

**Published:** 2025-08-11

**Authors:** Kangguo Li, Jiadong Wu, Ruixin Zhang, Yulun Xie, Zecheng Zhou, Qi Yin, Qi Chen, Jia Rui, Xuhua Guan, Zeyu Zhao, Tianmu Chen

**Affiliations:** 1 State Key Laboratory of Vaccines for Infectious Diseases, Xiang An Biomedicine Laboratory, State Key Laboratory of Molecular Vaccinology and Molecular Diagnostics, School of Public Health, Xiamen University, Xiamen, China; 2 Sichuan Provincial Comprehensive Clinical Center for Public Health, Jincheng Hospital, West China Hospital, Sichuan University, Chengdu, China; 3 Department of Acute Infectious Disease Control, Institute of Infectious Disease Prevention and Control, Hubei Provincial Center for Disease Control and Prevention, Wuhan, China; 4 Department of Epidemiology and Health Statistics, Xiangya School of Public Health, Central South University, Changsha, China

**Keywords:** Pertussis, Resurgence, Adult, Incidence, Disability-adjusted life years

## Abstract

**Background:**

Despite the high coverage of childhood vaccination, pertussis remains a significant global health challenge, with increasing adult cases attributed to waning immunity and enhanced diagnostic capability. This study quantified the global burden of pertussis in adults from 1990 to 2021 and evaluated the impact of the COVID-19 pandemic on disease trends.

**Methods:**

Using data from the Global Burden of Disease Study 2021, we assessed pertussis incidence and disability-adjusted life years (DALYs) in adults, stratified by age, sex, sociodemographic factors, and geographic regions. Temporal trends were analysed using joinpoint regression to detect significant changes and calculate the average annual percentage change (AAPC). An exponential smoothing state-space model with hierarchical forecast reconciliation was used to estimate the impact of the COVID-19 pandemic on pertussis burden.

**Results:**

Globally, the incidence rate of adult pertussis declined from 17.44 per 100,000 population in 1990 to 9.00 per 100,000 in 2019, and fell sharply to 2.70 per 100,000 by 2021. DALYs rates followed a similar trend. The burden was consistently highest in low Socio-demographic Index (SDI) countries, where the 2019 incidence rate was over four times that of high SDI countries (18.59 *vs*. 3.96 per 100,000). Between 1990 and 2019, incidence numbers increased in low SDI countries [AAPC: 0.63%; 95% confidence interval: 0.36%, 0.91%] and in older adults (AAPC > 0), despite falling incidence rates. From 2009 to 2019, incidence rates increased in 84 countries. During the COVID-19 pandemic, estimates based on the model indicated a 58.41% reduction in incidence and a 50.34% decrease in DALYs.

**Conclusions:**

Although the global incidence of adult pertussis has declined over the past three decades, a resurgence from 2009 to 2019, particularly in low-income regions and specific age groups, underscores the persistent challenges. The sharp decline during the COVID-19 pandemic highlights the importance of public health and social measures. These findings emphasise the need for targeted vaccination strategies and sustained surveillance to address regional disparities and prevent the resurgence of the disease.

**Graphical Abstract:**

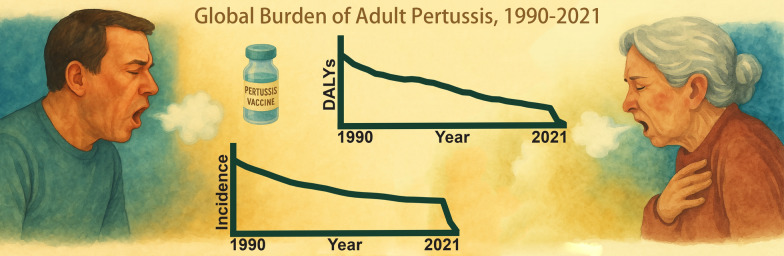

**Supplementary Information:**

The online version contains supplementary material available at 10.1186/s40249-025-01355-z.

## Background

Pertussis (whooping cough), a highly contagious respiratory infection caused by the *Bordetella pertussis* [1, 2], has historically been a significant cause of mortality in children. Although whole-cell (wP) vaccines were introduced in the mid-twentieth century, followed by acellular (aP) vaccines, which substantially decreased pertussis incidence, the disease has paradoxically resurged in recent decades in multiple regions, including Europe [3], America [4], East Asia [5], and Australia [6]. Notably, this resurgence was particularly pronounced in 2024, with reported cases in several countries exceeding those of the previous decade [3, 4, 7]. This resurgence is partly attributed to waning vaccine-derived immunity, underreporting, and evolutionary changes in the bacterium [1, 5, 8].

Pertussis increasingly affects adolescents and adults, challenging the long-held perception of the disease as being predominantly paediatric [9]. This shift has increased the median patient age. For instance, in Canada, the median age of patients with pertussis rose from 5 years in 1993 to 11 years during the 2000 outbreak [10]. Similarly, in Queensland, Australia, the median age increased from 12.6 years in 1997 to 54.5 years in 2007 [11]. This alteration in age distribution is believed to correlate with the widespread implementation of the diphtheria-tetanus-pertussis (DTP) vaccine through the WHO’s Expanded Program on Immunisation (EPI) [1]. While this initiative has effectively safeguarded younger populations, it has inadvertently rendered older, partially immunised, and unvaccinated individuals vulnerable.

Infants and unvaccinated individuals are at the highest risk for severe disease and complications, contributing to elevated mortality rates in these groups [1, 12]. In contrast, adults and older adults often present with mild or atypical symptoms, leading to frequent misdiagnoses and underreporting in clinical practice [13]. This issue is particularly pronounced in regions lacking advanced diagnostic techniques such as nucleic acid testing methods [14]. A modelling study suggested that the incidence of pertussis among adults aged ≥ 50 years in five Latin American countries may be approximately 100 times greater than that reported in the surveillance data [15]. Consequently, controlling pertussis remains a critical global health priority because of its persistent and growing burden, particularly among vulnerable populations such as older adults.

Using data from the Global Burden of Diseases (GBD) 2021 dataset, existing studies have emphasised the global burden of pertussis, noting a reduction in its incidence rate in recent decades [16–18]. However, the specific burden of this disease in adults is unclear. This study aimed to fill this gap by systematically analysing the burden of adult pertussis, focusing on its increasing incidence across age groups, sex, and World Bank geographical regions. We also examined the impact of the COVID-19 pandemic on worldwide pertussis incidence. By analysing temporal trends in adult patients with pertussis, this study aimed to provide a more accurate understanding of the disease and inform the development of effective global prevention and control strategies.

## Methods

### Data sources

The GBD 2021 provides comprehensive estimates of the global burden of 371 diseases and injuries across 204 countries and territories from 1990 to 2021. This analysis employed the latest epidemiological data and advanced, standardized methods. The methodological framework for mortality estimation has been detailed by the *GBD 2021 Diseases and Injuries Collaborators* [19, 20]. The GBD 2021 integrates various data sources, including vital registration systems, electronic health records, and epidemiological surveys through a systematic and structured process. Pertussis-specific metrics, such as incidence rates and disability-adjusted life years (DALYs), were estimated using advanced statistical modelling techniques. Mortality estimates were generated using a cause-of-death ensemble model that synthesises multiple data sources to enhance predictive accuracy. Non-fatal outcomes were modelled using DisMod-MR 2.1, a Bayesian meta-regression tool designed to ensure consistency among incidence, prevalence, and remission rates. To address data sparsity, spatiotemporal Gaussian process regression was used, leveraging spatial and temporal correlations to generate robust estimates in data-limited regions [19]. The uncertainty interval (*UI*) was calculated using 1000 Monte Carlo simulations, generating distributions of plausible outcomes represented by a 95% *UI* [19, 20].

To assess the burden of pertussis among adults aged ≥ 20 years, this study used data from the GBD 2021. As pertussis-related mortality is concentrated in infants and is negligible among our target population (adults aged ≥ 20 years) [18], our analysis focused on incidence rates and numbers, as well as DALYs rates and numbers, to assess disease burden in this demographic population. All data were obtained from the GBD Results Tool (https://www.healthdata.org/data-tools-practices/interactive-visual). To allow for a detailed age-specific analysis, we stratified the population into eight age groups: 20–24, 25–29, 30–34, 35–39, 40–44, 45–49, 50–54, and ≥ 55 years. This stratification enabled a more granular analysis of how the pertussis burden varies across different adult age groups, offering insights into the evolving epidemiology of pertussis in older adults.

The Sociodemographic Index (SDI) was used to assess the influence of health-related social and economic factors on the pertussis burden across regions and countries. The SDI is calculated as the geometric mean of three indicators: the total fertility rate for women under 25 years, mean education in adults aged 15 years and older, and lag-distributed income per capita. The values range from 0 (minimum development) to 1 (maximum development), allowing for the stratification of countries and territories into five categories: low (0–0.455), low-middle (0.456–0.608), middle (0.609–0.690), high-middle (0.691–0.805), and high SDI (0.806–1.000) quintiles [19]. We also applied the World Bank’s regional classification to further contextualise the results. This classification categorises countries into regions based on geographical proximity, encompassing six World Bank regions: East Asia and the Pacific, Europe and Central Asia, Latin America and the Caribbean, the Middle East and North Africa, South Asia, and sub-Saharan Africa [21]. Epidemiological data were linked to spatial datasets by assigning unique identifiers to each geographic location, which were subsequently mapped to ISO 3166-1 alpha-3 country codes to ensure compatibility with geographic datasets [21].

### Statistical analysis

The incidence and DALYs of adult pertussis were analysed according to sex and age group at the national, regional, and SDI levels. To evaluate temporal trends in pertussis incidence and DALYs, we employed joinpoint regression analysis to identify inflexion points (joinpoints) over the study period, dividing the data into distinct linear segments. The joinpoint regression model can be mathematically represented as

$$ln\left( {y_{i} } \right) = \beta_{0} + \beta_{1} \times year_{i}$$

where ${y}_{i}$ the expected incidence or DALYs value for $i$ observation, ${\beta }_{0}$ is the intercept, and ${\beta }_{1}$ is the slope coefficient corresponding to the annual trend. The maximum number of allowed joinpoints was set to five, and the optimal model was selected using a permutation test, which is a robust method for identifying significant changes in the temporal trends.

For each segment identified using the joinpoint model, the annual percentage change (APC) was calculated based on the estimated slope of the log-linear regression model. To summarise trends across predefined intervals (1990–1999, 1999–2009, 2009–2019, and 2019–2021), we computed the AAPC by taking a weighted average of the slope coefficients (on the logarithmic scale) from the underlying joinpoint regression model. The weight assigned to each segment corresponds to the number of years in the segment interval. The AAPC was calculated using the following formula:

$$AAPC = \left\{ {exp\left( {\frac{{\mathop \sum \nolimits_{i = 1}^{n} w_{i} b_{i} }}{{\mathop \sum \nolimits_{i = 1}^{n} w_{i} }}} \right) - 1} \right\} \times 100$$

where ${b}_{i}$ is the slope coefficient (i.e., the annual change in the natural logarithm of the outcome) for the $i$ segment, and ${w}_{i}$ is the length (in years) of the $i$ segment within the target interval. This formula yields a summary of the average annual percentage change over the specified period. Additionally, confidence intervals (*CI*) and statistical significance for APC and AAPC were derived using parametric methods based on the joinpoint regression model, assuming normality of residuals. All analyses were stratified by age, sex, and geographic region to explore the patterns of disease burden across subpopulations. A positive AAPC indicates an increasing trend, whereas a negative AAPC indicates a decreasing trend in pertussis incidence or DALYs over the specified interval.

To assess the potential impact of the COVID-19 pandemic on pertussis incidence, we used an exponential smoothing state-space model (ETS) to forecast pertussis cases in 2020 and 2021 [22]. Additionally, we constructed forecasts stratified by location, age, and sex to capture demographic and spatial heterogeneity. To ensure consistency across aggregation levels, base forecasts were reconciled using the ordinary least squares (OLS) method [23], which adjusts individual forecasts to align with hierarchical constraints based on the following formula:

$${\mathbf{Y}}_{t + h} = {\mathbf{SG\widehat{Y}}}_{t}$$

In this equation, the matrix ${\mathbf{Y}}_{t+h}$ represents the vector of reconciled forecasts. The matrix $\mathbf{S}$ transforms the disaggregated forecasts into aggregated forecasts by summing individual predictions according to the hierarchical structure. The matrix $\mathbf{G}$ modifies the base forecasts to minimise the overall forecasting error across the entire hierarchy.

The impact of COVID-19 was quantified by comparing the forecasted and observed pertussis incidence in 2020 and 2021. Specifically, we calculated both the absolute difference (observed minus predicted number) and relative difference (percentage reduction relative to the expected number) for each age group and the overall population. These deviations reflect the potential decrease in incidence and DALYs numbers attributable to public health and social measures (PHSMs), such as social distancing and changes in healthcare utilisation.

All statistical analyses were conducted using R 4.4.2 (Core Team, Vienna, Austria) and Joinpoint Regression Software Command 5.2.0 (National Cancer Institute, Bethesda, MD, USA). Data preprocessing and cleaning were performed using Python 3.10.8 (Python Software Foundation, Wilmington, DE, USA). Differences were considered statistically significant at a *P*-value of less than 0.05.

## Results

### Global burden and trends

In 1990, the estimated global number of pertussis cases among adults aged ≥ 20 years was 0.54 million (95% *UI*: 0.41, 0.68 million), corresponding to an incidence rate of 17.44 (95% *UI*: 13.36, 22.20) per 100,000 population. Over the next three decades, the global burden of adult pertussis showed an overall downward trend, albeit with fluctuations. By 2019, before the COVID-19 pandemic, this number had declined to 0.46 million (95% *UI*: 0.36, 0.59 million), with an incidence rate of 9.00 (95% *UI*: 6.96, 11.46) per 100,000 population. The number decreased sharply thereafter to 0.14 million (95% *UI*: 0.09, 0.21 million) and an incidence rate of 2.70 (95% *UI*: 1.81, 4.02) per 100,000 population by 2021 (Tables S1 and S2).

**Fig. 1:**
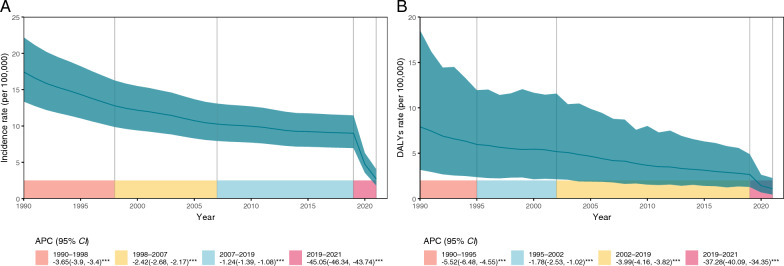
Joinpoint regression analysis of global pertussis incidence and disability-adjusted life years (DALYs) rates in adults aged ≥ 20 years from 1990 to 2021. **A** Estimated global incidence rate and annual percentage change (APC). **B** Estimated global DALYs rate and APC

Joinpoint regression analysis of the incidence rates identified four distinct phases. The rate declined from 1990 to 1998 (APC: −3.65%; 95% *CI:* −3.90%, −3.40%; *P* < 0.001), followed by slower declines from 1998 to 2007 (APC: −2.42%; 95% *CI:* −2.68%, −2.17%; *P* < 0.001), and from 2007 to 2019 (APC: −1.24%; 95% *CI:* −1.39%, −1.08%; *P* < 0.001). The sharpest reduction occurred between 2019 and 2021 (APC: −45.05%; 95% *CI:* −46.34%, −43.74%; *P* < 0.001) (Fig. 1A). Additionally, the incidence number decreased steadily from 1990 to 1999 (AAPC: −1.75; 95% *CI:* −1.93%, −1.56%; *P* < 0.001), and more slowly from 1999 to 2009 (AAPC: −0.34%; 95% *CI:* −0.52%, −0.16%; *P* < 0.001). However, joinpoint analysis showed an increasing trend in case numbers from 2006 to 2019 (AAPC: 0.38%; 95% *CI:* 0.26%, 0.50%; *P* < 0.001) (Table S1, Fig. S1).

Despite fluctuations in incidence numbers, the long-term trend for the DALYs was consistently downward. In 1990, the estimated rate of DALYs was 7.91 (95% *UI*: 3.17, 18.54) per 100,000 population. This rate decreased to 2.66 (95% *UI*: 1.29, 4.89) per 100,000 population by 2019 and fell further to 1.10 (95% *UI*: 0.45, 2.27) per 100,000 population by 2021 (Table S1). Joinpoint analysis also showed four phases of decline in the DALYs rate, with joinpoints observed in 1995, 2002, and 2019, all with APC values less than 0 and *P* < 0.001 (Fig. 1B, Fig. S1). The rate decreased from 1990 to 1995 (APC −5.52%; 95% *CI:* −6.48%, −4.55%; *P* < 0.001) and from 1995 to 2002 (APC −1.78%; 95% *CI:* −2.53%, −1.02%; *P* < 0.001). The decline then accelerated from 2002 to 2019 (APC −3.99%; 95% *CI:* −4.16%, −3.82%; *P* < 0.001), followed by the most dramatic decrease from 2019 to 2021 (APC −37.28%; 95% *CI:* −40.09%, −34.35%; *P* < 0.001) (Fig. 1B).

From 1990 to 2019, trends in pertussis incidence were similar for males and females, with an AAPC of approximately −0.54% for both sexes (Table S2). During this period, however, the reduction in DALYs was greater in males (AAPC −2.16%; 95% *CI:* −2.36%, −1.96%; *P* < 0.001) than in females (AAPC −1.59%; 95% *CI:* −1.85%, −1.33%; *P* < 0.001). Following the sharp declines between 2019 and 2021, these sex differences were largely eliminated (Table S3). Global incidence rates were 2.64 per 100,000 population in females and 2.77 per 100,000 population in males, and DALYs rates were 1.06 per 100,000 population in females and 1.14 per 100,000 population in males (Tables S2, S3).

### Variation by age group

Between 1990 and 2019, trends in the incidence and rate varied by age (Fig. S2, Fig. S3). The number of cases significantly declined in adults younger than 45 years (all *P* < 0.05), with AAPCs ranging from −0.31 to −1.14%. Whereas the trend was stable for 45–49 years (AAPC: 0.03%; 95% *CI:* −0.23%, 0.29%; *P* = 0.83), 50–54 years (AAPC: −0.07%; 95% *CI:* −0.19%, 0.05%; *P* = 0.25), and ≥ 55 years (AAPC: −0.14%; 95% *CI:* −0.48%, 0.19%; *P* = 0.40) (Table S2). This stability in older groups reflects a significant increase in case numbers between 2009 and 2019 for 45–49 years (AAPC: 0.81%; 95% *CI:* 0.59%, 1.03%; *P* < 0.001), 50–54 years (AAPC: 1.58%; 95% *CI:* 1.44%, 1.72%; *P* < 0.001), and ≥ 55 years (AAPC: 1.66%; 95% *CI:* 1.82%, 2.51%; *P* < 0.001). This occurred despite consistently declining incidence rates across all age groups during the same period, with a significant reduction in ≥ 55 years (AAPC: −1.37%; 95% *CI:* −1.88%, −0.85%; *P* < 0.001) (Table S4).

Among the various age groups, the highest incidence rate was recorded in the 40–44 year age group. In 2019, the incidence rate for the 40–44 year age group was 12.98 (95% *UI*: 10.03, 16.53) per 100,000 population, which declined to 3.85 (95% *UI*: 2.56, 5.77) per 100,000 population by 2021. Conversely, those aged ≥ 55 years exhibited the lowest incidence rate, with figures of 4.87 (95% *UI*: 3.77, 6.20) per 100,000 population in 2019 and 1.27 (95% *UI*: 0.84, 1.91) per 100,000 population in 2021 (Fig. S2). From 1990 to 2019, the incidence rates across all age groups demonstrated a consistently declining trend, with AAPCs ranging from −1.70% to −2.64%. Between 2019 and 2021, all age groups experienced a substantial reduction in incidence rate, with AAPCs ranging from −42.10 to −48.65% (*P* < 0.001 for all) (Table S2).

The trends for DALYs mirrored those for incidence (Fig. S4, Fig. S5). From 2009 to 2019, the decline in DALYs rates was more pronounced with increasing age, from −2.49% (95% *CI:* −3.05%, −1.92%; *P* < 0.001) in the 20–24 year age group to −4.35% (95% *CI:* −4.47%, −4.23%; *P* < 0.001)in those aged ≥ 55 year. This decline accelerated considerably between 2019 and 2021, with AAPCs ranging from −35.14 to −38.93% across age groups (Table S3, Fig. S5).

### Variation by the SDI

The adult pertussis incidence rate was inversely related to SDI throughout the study period. In 2019, the incidence rate remained inversely related to the SDI: 18.59 (95% *UI*: 14.26, 23.67) per 100,000 population in low-SDI countries, more than four times the rate of 3.96 (95% *UI*: 3.04, 5.10) per 100,000 population in high-SDI countries. Although every region experienced further reductions by 2021, reaching 8.46 (95% *UI*: 6.07, 11.27) and 0.39 (95% *UI*: 0.25, 0.64) per 100,000, respectively, relative disparities persisted (Fig. S6).

Between 1990 and 2019, the incidence rates declined across all SDI quintiles, with AAPCs ranging from −1.88 to −3.60% (all *P* < 0.001) (Table S2). However, the trend in the absolute number of incidences differed substantially by the development level (Fig. S7). Incidence numbers increased in low-SDI countries (AAPC: 0.63%; 95% *CI:* 0.36%, 0.91%; *P* < 0.001), particularly between 1990 and 2009. In contrast, incidence numbers were stable in middle SDI countries (AAPC: −0.05%; 95% *CI:* −0.39%, 0.29%; *P* = 0.76) and decreased in high-middle and high SDI quintiles (−2.35% and −1.81%, respectively) (Table S2).

A similar pattern was observed for the DALYs rates, which also showed a clear inverse gradient with the SDI (Fig. S6). Between 2019 and 2021, the DALYs rate fell from 10.77 (95% *UI*: 4.58, 20.96) per 100,000 to 4.62 (95% *UI*: 1.79, 9.70) per 100,000 in low SDI settings, and from 0.08 (95% *UI*: 0.05, 0.15) per 100,000 to 0.02 (95% *UI*: 0.01, 0.03) per 100,000 in high SDI settings. From 1990 to 2019, the AAPC decline in DALYs rates was most pronounced in the high-middle and middle SDI countries (Table S3).

The DALYs rate exhibited a similar pattern, falling from 10.77 (95% *UI*: 4.58, 20.96) per 100,000 population to 4.62 (95% *UI*: 1.79, 9.70) per 100,000 in low-SDI settings between 2019 and 2021, compared with a decline from 0.08 (95% *UI*: 0.05, 0.15) per 100,000 population to 0.02 (95% *UI*: 0.01, 0.03) per 100,000 population in high-SDI countries (Fig. S6).

### National burdens and trends

**Fig. 2:**
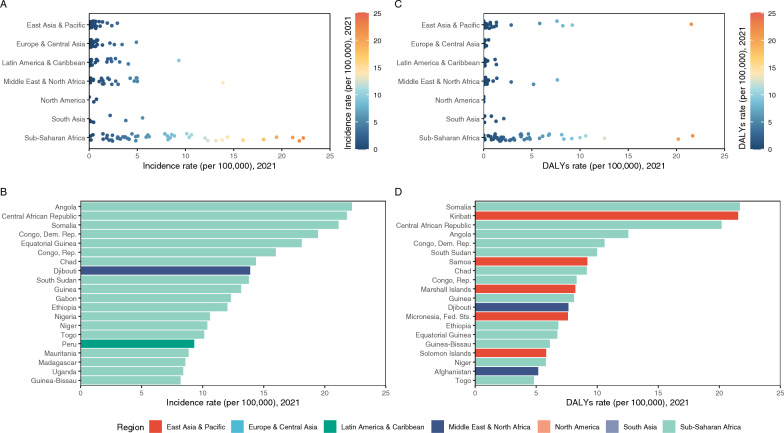
Distribution of pertussis incidence and disability-adjusted life years (DALYs) rates among adults aged ≥ 20 years in 2021. **A** Estimated pertussis incidence rate in various countries and territories in 2021. **B** The top 20 countries and territories by pertussis incidence rate in 2021. **C** Estimated pertussis DALYs rate in various countries and territories in 2021. **D** Top 20 countries and territories by pertussis DALYs rate in 2021

Regional disparities in adult pertussis incidence and DALYs rates were notable in 2021, with sub-Saharan Africa consistently bearing the highest regional incidence and DALYs rates worldwide (Fig. 2A, C). In 2021, this burden was concentrated in six countries: Angola, the Central African Republic, Somalia, the Democratic Republic of Congo, Equatorial Guinea, and the Republic of Congo (Fig. 2D). Although the DALYs rate in this region nearly halved from 1990 to 2019, decreasing from 21.14 (95% *UI*: 8.32, 42.88) per 100,000 population to 10.21 (95% *UI*: 4.26, 19.70) per 100,000 population, the total number of DALYs increased steadily over the same period, with an AAPC of 0.49% (95% *CI:* 0.20%, 0.79%; *P* = 0.001). This contrasts with the declining number of DALYs in all other super regions (Table S3).

**Fig. 3:**
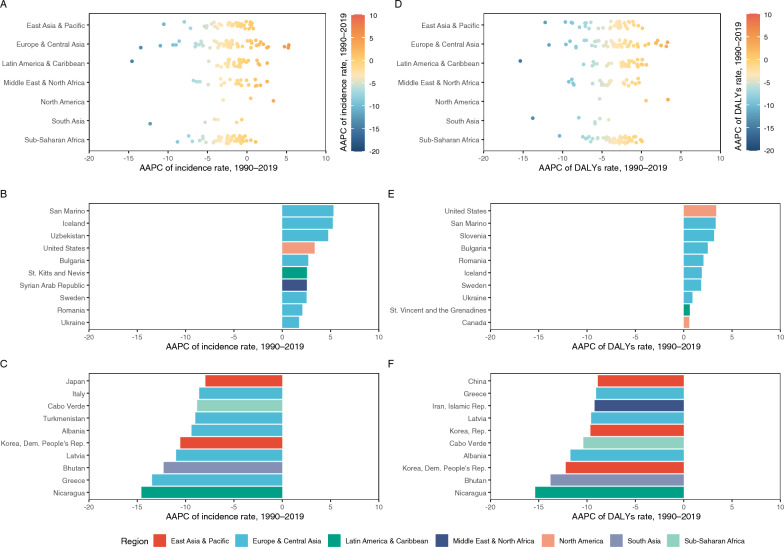
Distribution showing the adjusted annual percentage change (AAPC) in pertussis incidence and disability-adjusted life years (DALYs) rates in adults aged ≥ 20 years from 1990 to 2019. **A** Estimated AAPC of incidence rate across various countries and territories. **B** Top 10 countries and territories by the AAPC of the pertussis incidence rate. **C** The bottom 10 countries and territories by AAPC of the pertussis incidence rate. **D** Estimated AAPC of the DALYs rate across various countries and territories. **E** Top 10 countries and territories by the AAPC of pertussis DALYs rate. **F** The bottom 10 countries and territories by AAPC of the pertussis DALYs rate

Despite an overall global decline, the incidence rates at the national level increased in many countries between 1990 and 2019, particularly in North America, Africa, Australia, Central Asia, and Southeast Asia (Fig. 3A). Of the 204 countries and territories analysed, 37 had a rising incidence rate, with AAPCs greater than zero (Fig. 3B, C). Notably, the most rapid increases were observed in Iceland (AAPC: 5.23; 95% *CI:* 4.02, 6.47; *P* < 0.001) and San Marino (AAPC: 5.30%; 95% *CI:* 4.83%, 5.78%; *P* < 0.001) (Fig. 3A, B). Fewer countries (15 of 204) recorded an increase in the DALYs rates over this period (Fig. 3D, E, F). The United States exhibited the highest growth rate, with an AAPC of 3.35% (95% *CI:* 2.56%, 4.15%; *P* < 0.001), followed by San Marino (AAPC: 3.30%; 95% *CI:* 2.60%, 4.00%; *P* < 0.001) and Slovenia (AAPC: 3.14%; 95% *CI:* 2.25%, 4.00%; *P* < 0.001) (Fig. 3E).

A global resurgence of pertussis was evident from 2009 to 2019, with AAPCs were positive in Europe & Central Asia (AAPC: 3.70%; 95% *CI:* 2.77%, 4.65%; *P* < 0.001), Middle East & North Africa (AAPC: 4.15%; 95% *CI:* 3.52%, 4.79%; *P* < 0.001), and Latin America & Caribbean (AAPC: 5.43%; 95% *CI:* 5.07%, 5.79%; *P* < 0.001) (Table S6). At the national level, 84 countries reported increasing incidence rates, and 38 reported increasing DALYs rates. Brazil experienced one of the most pronounced increases, with its incidence rate rising from 2.37 (95% *UI*: 1.77, 3.13) per 100,000 population to 11.13 (95% *UI*: 8.60, 14.12) per 100,000 population, with corresponding AAPCs of 15.64% (95% *CI:* 14.72%, 16.58%; *P* < 0.001) (Supplement 2).

### Impact of COVID-19

**Fig. 4:**
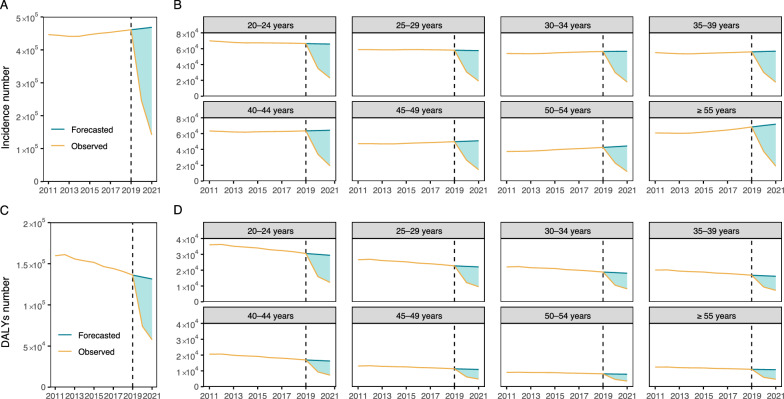
Impact of the COVID-19 pandemic on pertussis incidence and disability-adjusted life years (DALYs) numbers across different age groups in adults aged ≥ 20 years from 2020 to 2021. **A** Total observed and forecasted global pertussis incidence number. **B** Observed and forecasted global incidence number of pertussis in various adult age groups. **C** Total observed and forecasted global pertussis DALYs number. **D** Observed and forecasted global DALYs number across various adult age groups

The COVID-19 pandemic coincided with a notable global decline in the incidence of both pertussis and DALYs between 2019 and 2021 (Table S2). Using a time-series model based on pre-pandemic data, we forecasted 464,769 and 468,475 cases in 2020 and 2021, respectively. The observed number of cases was 58.4% lower than the forecast (Fig. 4A). The magnitude of this reduction varied by age; it was most pronounced in adults aged ≥ 55 years (−60.42%) and smallest in those aged 20–24 years (−56.06%) (Fig. 4B). In parallel, the predicted global DALYs for 2020 and 2021 were estimated to be 133,813 and 133,469, respectively, indicating a decrease of 50.34% (Fig. 4C). The most significant reduction in DALYs was also observed in the 20–24 age group (−52.52%), whereas the 50–54 age group experienced a smaller decline of 48.58% (Fig. 4D).

**Fig. 5:**
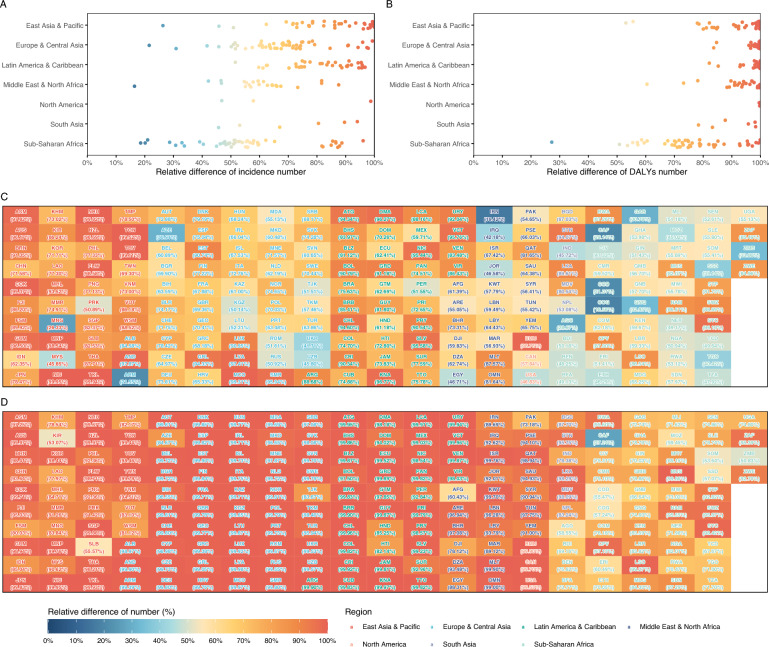
Impact of the COVID-19 pandemic on pertussis incidence and disability-adjusted life years (DALYs) numbers across different countries in adults aged ≥ 20 years from 2020 to 2021. **A** Reduction in incidence across various regions. **B** Reduction in DALYs numbers across multiple regions. **C** Reduction in incidence across various countries and territories. **D** Reduction in the number of DALYs across multiple countries and territories. The connection between ISO3 country codes and country names is available from the World Bank (https://datahelpdesk.worldbank.org/knowledgebase/articles/906519-world-bank-country-and-lending-groups)

The impact on pertussis incidence varied considerably among countries. Between 2019 and 2021, the observed incidence in 175 countries and territories was more than 50% lower than the forecasted trend between 2020 and 2021. The largest reductions, exceeding 99%, occurred in several small island nations, including Niue (99.95%), Tokelau (99.94%), Nauru (99.82%), Tonga (99.52%), Cook Islands (99.37%), Monaco (99.31%), and Tuvalu (99.11%). In contrast, the smallest declines were observed in Iran (16.42%) and Congo (18.56%), and many countries with smaller reductions were in sub-Saharan Africa. Among the larger populations, the reduction in incidence was greater in China (69.30%), Brazil (75.63%), and Indonesia (62.35%), than in the United States (46.83%) and India (45.72%) (Fig. 5A, C).

A similar widespread reduction was observed for the DALYs, although the magnitude varied (Fig. 5B, D). The Central African Republic recorded the smallest decline at 27.24% compared to the forecast. In most other countries, the reductions were substantial. For example, the observed DALY burden in the United States and India was 99.53% and 78.38% lower than forecasted, respectively, whereas in other countries, the reductions ranged from 50.83% to more than 99.97% (Fig. 5B, D).

## Discussion

Our comprehensive analysis revealed a significant, yet complex, long-term decline in the global burden of adult pertussis between 1990 and 2021. This study highlights the dynamic nature of pertussis transmission by examining its spatiotemporal distribution, demographic patterns, and the impact of major socioeconomic events. These findings underscore the sensitivity of pertussis transmission to societal dynamics and acute global disruptions such as the COVID-19 pandemic. The international scope of this study provides crucial data supporting targeted public health strategies and emphasises the ongoing need for continuous surveillance and tailored PHSMs, particularly in the adult and elderly populations.

The overall trajectory of adult pertussis globally showed a marked decline between 1990 and 2021, with the number of DALYs decreasing from 0.24 million to 0.06 million and the incidence falling from 0.54 million to 0.14 million cases. This favourable long-term trend is mainly attributable to the widespread implementation and sustained impact of the global childhood EPI [24]. These programs have reduced overall transmission, thereby affording indirect protection to the adult population by diminishing their exposure to *B. pertussis* [25]. However, this optimistic global narrative is complicated by periods of fluctuations and regional disparities. A critical observation from our analysis was the increase in pertussis incidence number, specifically, between 2009 and 2019. This resurgence was not uniformly distributed; regions such as the Middle East and North Africa, Latin America, and the Caribbean witnessed incidence rates in 2019 that approached or surpassed those recorded in 2000 (Fig. S7). Such patterns of localised resurgence, occurring against a backdrop of overall global decline, underscore the complex, evolving epidemiology of pertussis and the persistent challenges in achieving consistent, widespread disease control.

Several interacting factors may have underpinned the observed increase from 2009 to 2019. First, waning immunity, whether vaccine-induced or naturally acquired, is a well-documented phenomenon in pertussis [9]. Published data indicate that immunity from pertussis infection after 4–20 years, whereas protection from vaccination typically wanes after 4–12 years [26]. Consequently, adults vaccinated in childhood or adolescence decades prior, particularly in settings where adult booster vaccinations are not routinely administered, may possess insufficient protection, rendering them susceptible to infection and capable of transmitting *B. pertussis*. This vulnerability is likely exacerbated by regional differences in primary vaccination coverage, specific strategies employed, and the general absence of widespread and systematically implemented adult booster programs worldwide [24, 27, 28].

Second, the evolution of *B. pertussis*, including the emergence and circulation of genetically diverse strains, presents another significant challenge. These adaptations may enable immune evasion and potentially reduce the effectiveness of existing vaccines, thereby facilitating increased infection rates even in previously vaccinated populations. For instance, an analysis of *B. pertussis* isolates in Norway from 1996 to 2019 revealed a shift towards strains with pertussis toxin (*ptxA1*), pertussis toxin promoter (*ptxP3*), and pertactin (*prn2*) alleles, with 16% of isolates showing *prn* gene mutations [29]. Similar trends in pathogen evolution, possibly driven by vaccine pressure from aP vaccines and booster doses, have been observed in Japan, the United States, Australia, and China [30–32]. Such antigenic drift may undermine vaccine efficacy and pose challenges to sustained pertussis control efforts [33].

Third, advances in public health surveillance and diagnostic technologies have enhanced case ascertainment. The increased adoption of highly sensitive techniques, such as polymerase chain reaction (PCR) assays, coupled with gradually improving diagnostic capabilities in developing countries, has facilitated better detection of pertussis in adults [34]. While these advancements improve the accuracy of disease burden estimates, they may also contribute to an apparent rise in incidence, as previously undetected or misdiagnosed cases are now properly identified [8]. Thus, the observed rise in reported cases during this period likely reflects both a genuine increase in transmission and improved detection of previously unrecognised infections in the population.

Fourth, demographic shifts may present another layer to this phenomenon, potentially explaining the apparent paradox where the global crude incidence rate rose while age-specific rates declined. As global populations age, a larger proportion of individuals moves into the middle-adulthood age brackets. Our analysis identified these specific age groups as having a persistently high incidence burden. Therefore, if the proportion of the population in middle or older age groups—which may have a higher baseline incidence—increased over this decade, it could mathematically drive the overall crude incidence rate upward, even as rates within each specific age bracket were falling [35].

The national-level trends further illustrate this complexity, with the United States presenting a particularly striking case. Contrary to the global trend of decreasing DALYs, the United States experienced the most significant growth in its DALYs rate from 1990 to 2019. This notable increase is likely multifactorial. It may be partly linked to the switch from wP to aP vaccines in the 1990s, as aP vaccines may induce a less durable immune response, contributing to a larger susceptible adult population over time [26]. Concurrently, heightened awareness and the widespread adoption of sensitive PCR diagnostic methods in the United States have almost certainly led to better case ascertainment in adults, who often present with atypical symptoms that were previously underdiagnosed [34]. This combination of factors could synergistically contribute to the observed rise in disease burden.

Age-specific patterns further highlight the substantial disparities and evolving vulnerabilities. Our study noted an increase in incidence within specific adult age groups, namely, 30–34, 45–49, and 50–54 years. This trend is particularly evident in lower-income regions, including sub-Saharan Africa, the Middle East, and North Africa. These age-specific surges likely reflect the cumulative effects of waning immunity from childhood vaccination. This is compounded by reduced opportunities for natural immune boosting in adulthood, attributable to the decreased circulation of *B. pertussis* following successful childhood EPI vaccination. These epidemiological trends necessitate a critical reassessment of current immunisation strategies, particularly those focusing on the adult population. While childhood vaccination programs have substantially reduced the burden of vaccine-preventable diseases in young children [16], there is a growing concern about adult vulnerability. Increasing evidence suggests that immunity against pertussis wanes over time. In response, some high-SDI countries, such as Canada and the United States [36, 37], have implemented recommendations for repeated tetanus, diphtheria, and aP vaccinations every 10 years following an adolescent booster dose. This strategy is immunogenic and well tolerated [38, 39]; however, the adoption of pertussis booster vaccinations among adults remains relatively low in many regions, including parts of the Asia-Pacific [40]. To address this gap, targeted efforts should prioritise high-risk groups, such as individuals with chronic respiratory conditions, including chronic obstructive pulmonary disease and asthma [40], who are more susceptible to pertussis and its complications. Improving disease awareness among the public and healthcare providers, coupled with the establishment of robust adult vaccination registries, could significantly improve vaccine coverage and support healthy aging.

Socioeconomic factors, particularly healthcare economic conditions and resource availability are pivotal determinants of the burden of pertussis across diverse populations. Existing evidence indicates that low-income countries often face persistently high incidence rates [16]. Our analysis substantiates this finding, revealing that regions with lower socioeconomic development bear disproportionately high burdens. For instance, in 2019, within the low-SDI quintile, the incidence rate reached 18.59 per 100,000 population, and the DALYs rate was notably high at 10.77 per 100,000 population, underscoring this disparity. Further illustrating this trend, between 1990 and 2019, high-middle SDI and high-SDI nations experienced significant declines in incidence numbers, but low-SDI countries witnessed an increase. This divergence highlights the challenges faced by nations with less developed economies in controlling disease transmission, often due to limitations in medical infrastructure, insufficient healthcare resource allocation, and barriers to accessing routine immunisation services and advanced diagnostics [41]. This situation is particularly acute in regions such as sub-Saharan Africa, where chronic under-resourcing contributes to a persistently elevated disease burden.

The COVID-19 pandemic has profoundly altered the epidemiology of infectious diseases [42]. Our findings showed a sharp 58.41% decline in reported adult pertussis incidence during the pandemic, consistent with Zhang et al.’s study [18]. These findings strongly suggest that COVID-19-related PHSMs effectively disrupted pertussis transmission [43, 44]. However, the pandemic's impact was highly heterogeneous. This decline was most pronounced in those aged ≥ 55 years, likely because of stricter adherence to protective measures in this group [45]. Geographically, isolated islands (Niue, Tokelau, and Nauru) showed the most significant decrease, but populous countries like China and Brazil also showed significant, albeit varied, decreases, and sub-Saharan African regions exhibited a much smaller reduction, possibly reflecting limitations in surveillance, control capabilities, or the extent of PHSMs implementation and adherence.

Nowhere was this heterogeneity more striking than in the United States, where incidence fell by 46.83% while DALYs plummeted by an astonishing 99.53%. This vast discrepancy suggests the pandemic environment had a disproportionate effect on preventing or recording severe, debilitating pertussis. This is likely attributable first to a combination of protective effects and diagnostic shifts; stricter adherence to PHSMs among high-risk groups selectively averted severe outcomes [45]. Concurrently, the intense focus on the pandemic may have led to misdiagnosis, where severe pertussis cases were under-ascertained as healthcare resources were reallocated [13]. Furthermore, the sheer scale of the pandemic's mortality impact must be considered a crucial factor. The COVID-19 pandemic caused a substantial loss of life that significantly shortened life expectancy, with the burden falling overwhelmingly on the elderly; for example, a systematic analysis of 18 European countries found that approximately 90% of the 16.8 million person-years of life lost from 2020 to 2022 occurred in individuals aged 65 and over [46]. This profound mortality within the very demographic most susceptible to severe pertussis outcomes inherently shrank the population pool that could contribute to the pertussis DALYs burden, thus contributing to its precipitous fall.

This study has certain limitations inherent to global burden analysis and epidemiological research. First, the GBD estimates were derived from mathematical modelling, the accuracy of which depends heavily on model assumptions, parameters, and the completeness and quality of input data. For instance, adult pertussis remains notoriously challenging to diagnose clinically because of its often-atypical presentation, potentially leading to significant underdiagnosis and underreporting even before GBD modelling adjustments; the extent of residual underestimation may vary. Consequently, additional field surveillance data are crucial for validating and refining the model estimates. Second, this study lacked granular data on several important covariates. These include detailed breakdowns of vaccination coverage (especially for adult boosters and the type of vaccine used, e.g. wP *vs.* aP and timing of transitions), specifics of country-level control measures, and comprehensive pathogen strain characteristics across all regions and periods of the study. The absence of these data constrained our ability to conduct a more thorough quantitative analysis of their influence on the observed disease burden. Over the extended study period, changes in pertussis case definitions or surveillance system sensitivity in various countries might have affected data consistency despite GBD standardisation efforts. Future studies should aim to incorporate and quantify the effects of these factors. Finally, although we included initial post-pandemic data, the COVID-19 pandemic is expected to have a complex, long-term impact on pertussis epidemiology, including potential shifts in population immunity, health-seeking behaviours, and surveillance practices. Current data remain insufficient to fully elucidate these long-term effects, underscoring the need for continued research.

## Conclusions

Our comprehensive analysis of adult pertussis cases from 1990 to 2021 revealed a complex epidemiological landscape. While the global incidence and DALYs showed an overall decline, this favourable trend was punctuated by significant regional resurgences, particularly in low-income regions. We attribute these increases to factors such as waning vaccine-derived immunity, evolutionary changes in *B. pertussis*, and enhanced diagnostic capabilities. The transient reduction in reported cases during the COVID-19 pandemic further highlights the sensitivity of pertussis transmission to PHSMs. These insights are crucial for informing targeted public health strategies and emphasising the need for tailored vaccination programmes and robust surveillance systems for adults, especially in resource-constrained settings.

## Supplementary Information

Supplementary material 1: Fig. S1-7; Table S1-7.

Supplementary material 2. Number, rate, and adjusted annual percentage change (AAPC) of pertussis incidence and Disability-adjusted life years (DALYs) among adults aged over 20 years across various countries and territories from 1990 to 2021, with estimates based on the joinpoint regression model.

## Data Availability

All data and R codes are available in the GitHub repository (https://github.com/xmusphlkg/adult_pertussis).

## Abbreviations

aP: Acellular pertussis
AAPC: Annual average percentage change
APC: Annual percentage change
CFR: Case fatality rate
*CI*: Confidence interval
DTP: Diphtheria-tetanus-pertussis
DALYs: Disability-adjusted life years
EPI: Expanded Program on Immunisation
ETS: Exponential smoothing state-space
GBD: Global Burden of Disease
OLS: Ordinary least squares
PHSMs: Public health and social measures
SDI: Sociodemographic index
UI: Uncertainty interval
wP: Whole-cell pertussis
