# Supplementary material for: Global, regional, and national burdens of pertussis among adults: a systematic analysis of age-specific trends using Global Burden of Diseases 2021 data
Source: Infect Dis Poverty. 2025 Aug 11;14:85. doi: 10.1186/s40249-025-01355-z (PMC12337552; doi:10.1186/s40249-025-01355-z)
Supplement: Supplementary file 1 — Supplementary material 1: Fig. S1-7; Table S1-7. [file 40249_2025_1355_MOESM1_ESM.docx]

**Supplementary Information for**

**Global, regional, and national burdens of pertussis among adults: a systematic analysis of age-specific trends using Global Burden of Diseases 2021 data**

*Kangguo Li, Jiadong Wu, and Ruixin Zhang et al.*


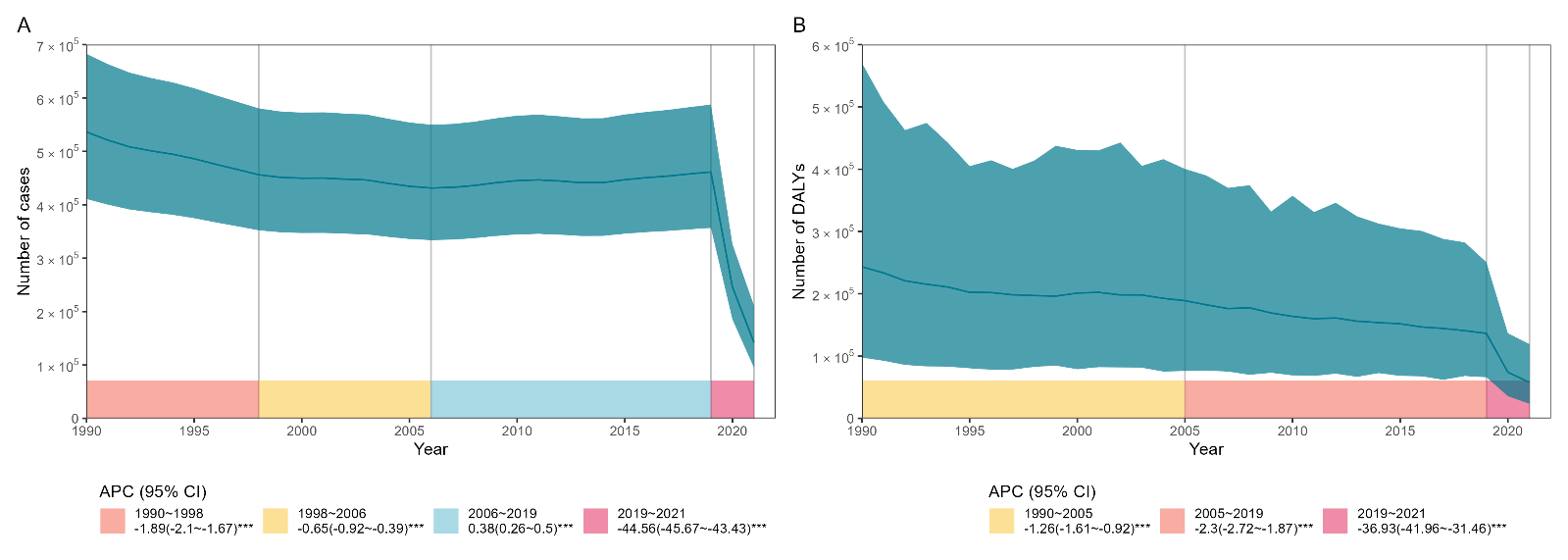


Fig.S1. Global annual percentage change (APC) in pertussis incidence and disability-adjusted life years (DALYs) number for adults aged over 20 years, 1990-2021. (A) APC in pertussis incidence with 95% confidence intervals; (B) APC in DALYs with 95% confidence intervals (*CI*).


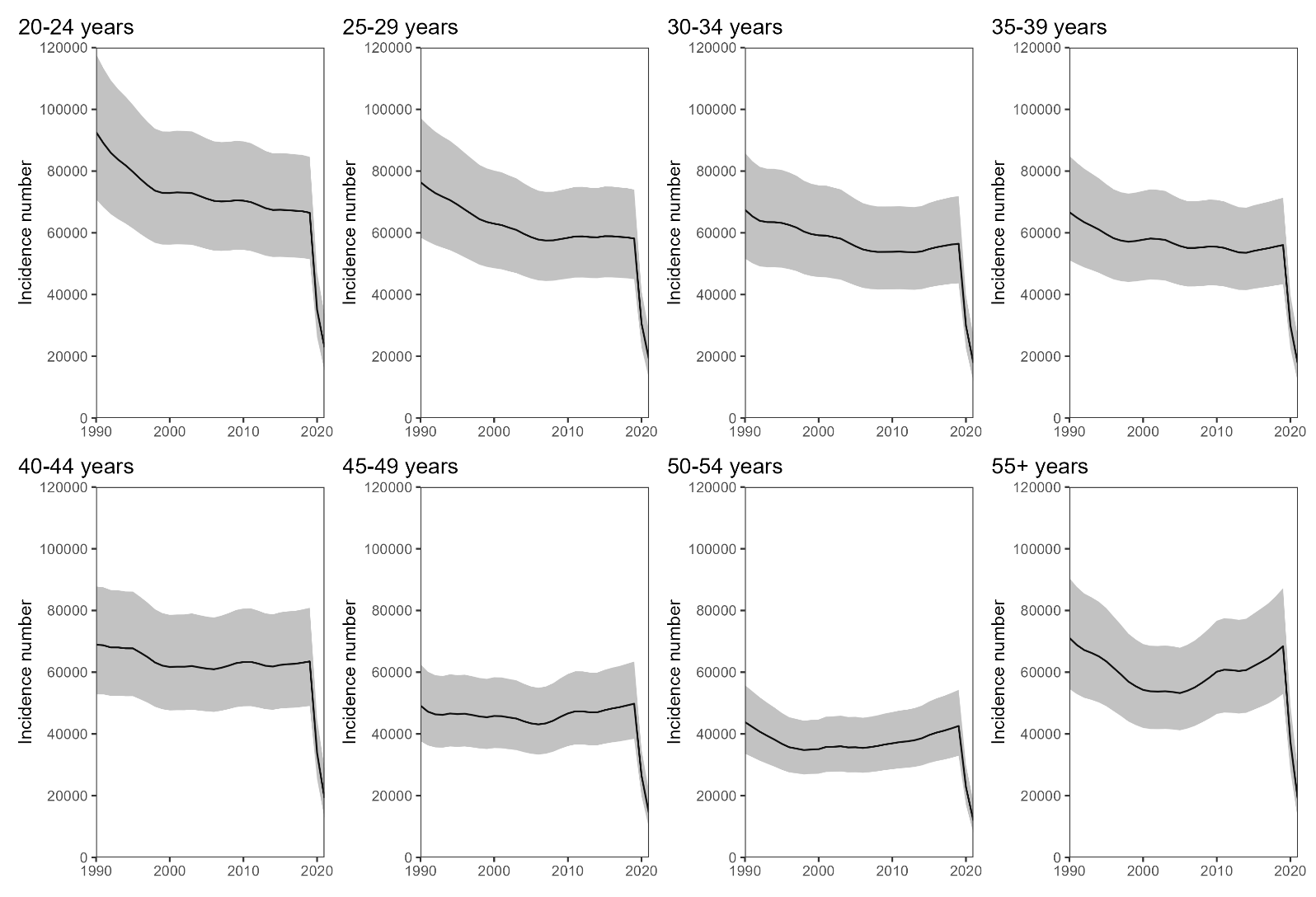


Fig. S2. Incidence numbers of pertussis across varied age groups from 1990 to 2021.


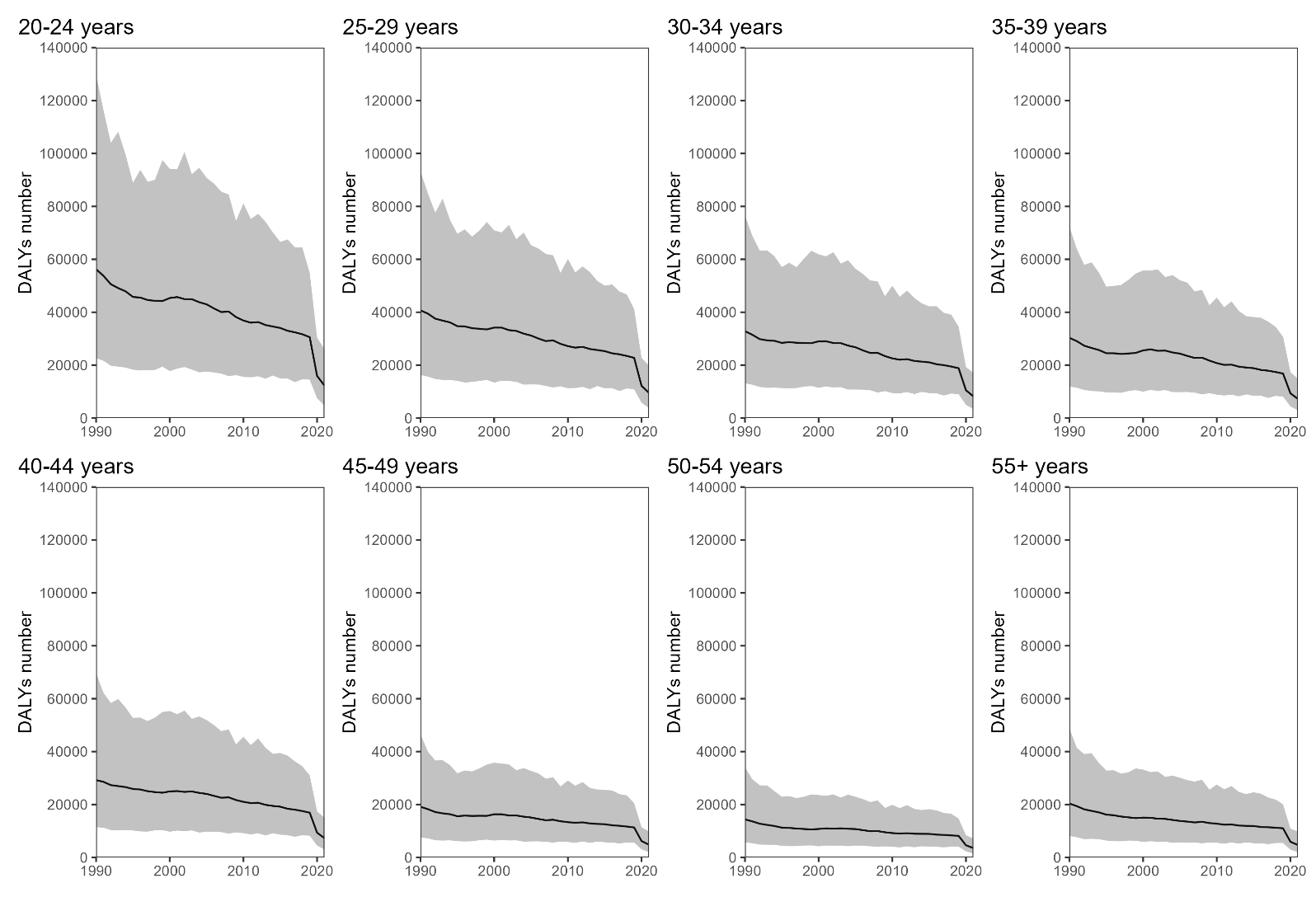


Fig.S3. Disability-adjusted life years (DALYs) number for pertussis across varied age groups from 1990 to 2021.


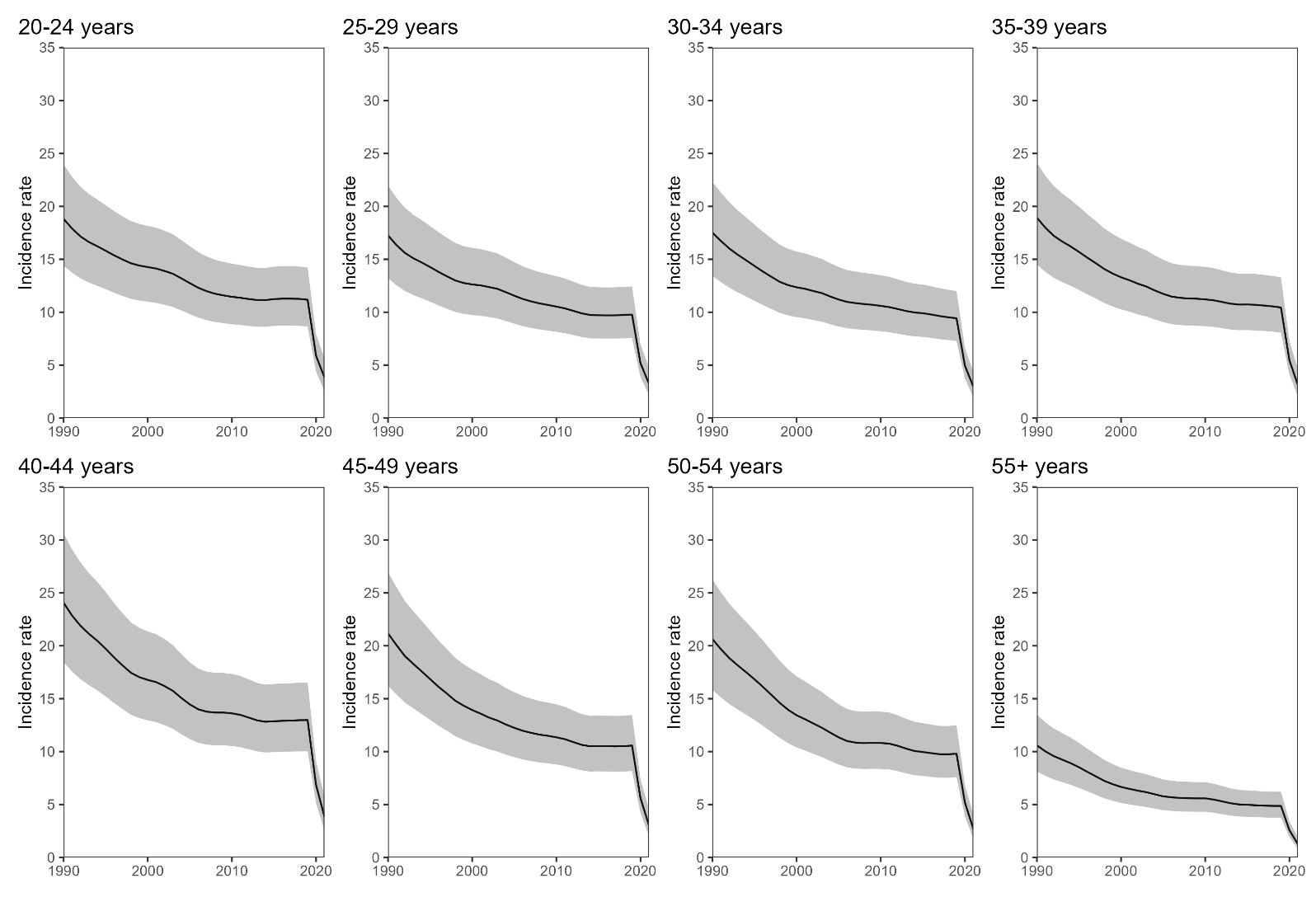


Fig.S4. Incidence rates of pertussis across varied age groups from 1990 to 2021.


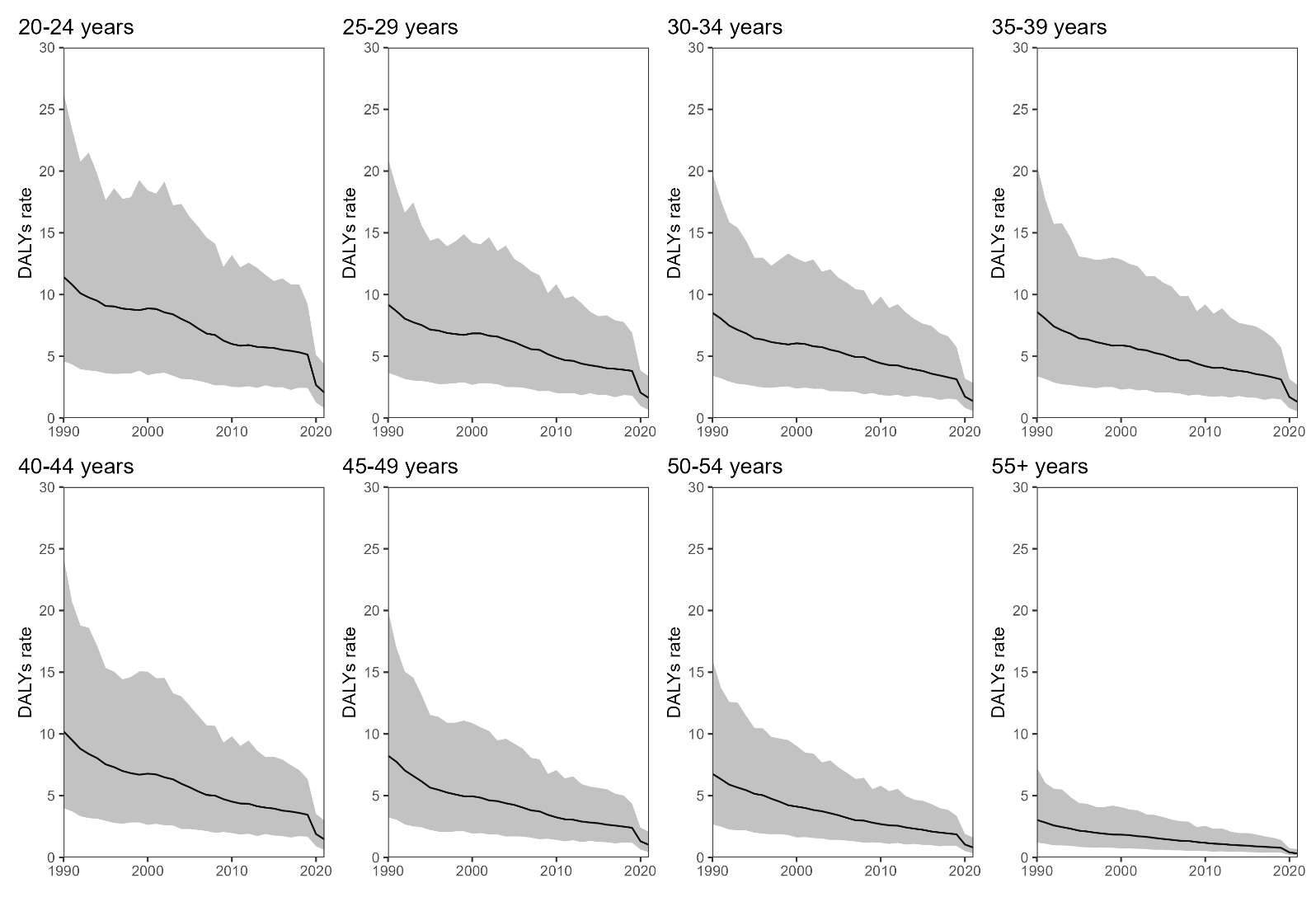


Fig. S5. Disability-adjusted life years (DALYs) rates of pertussis across varied age groups from 1990 to 2021.
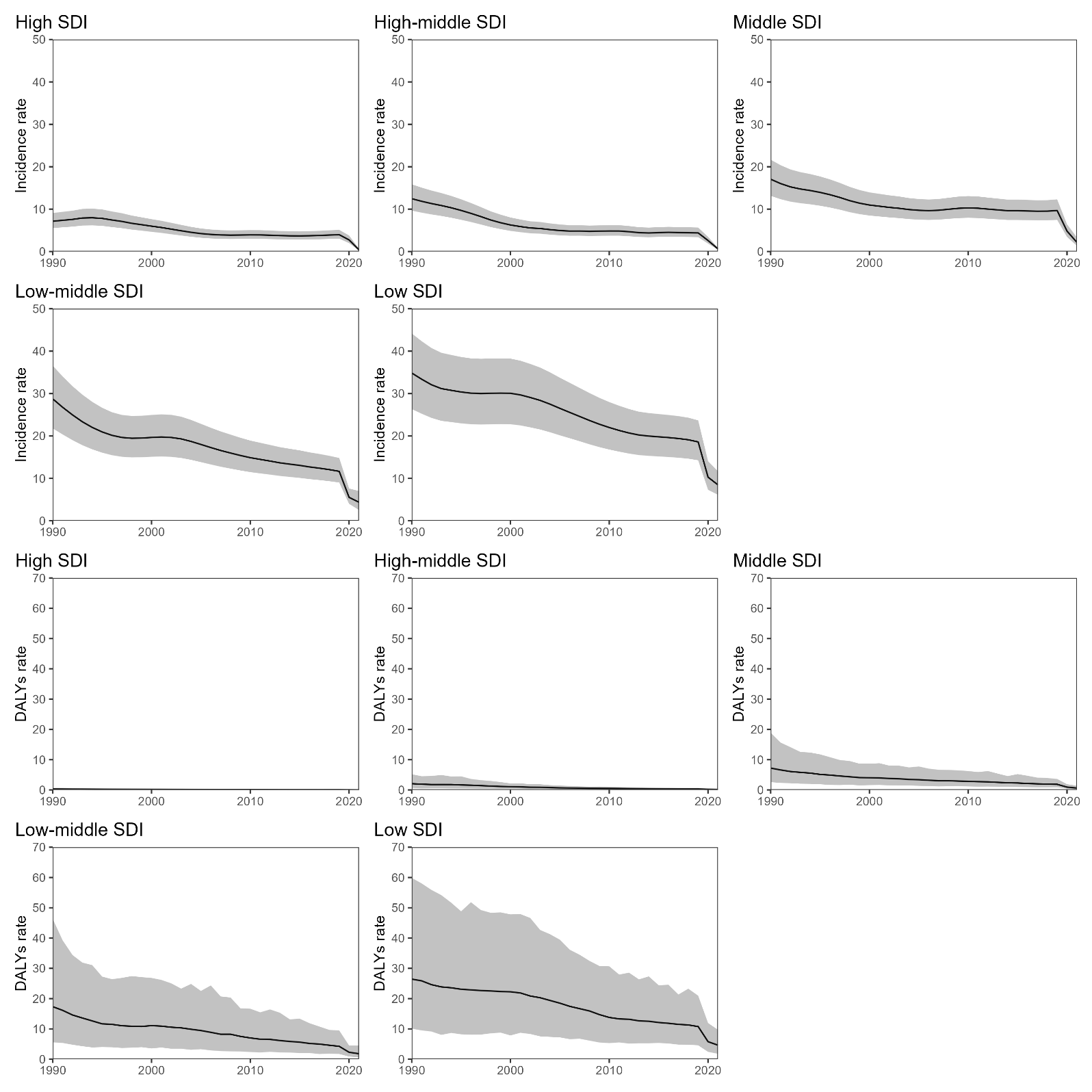


Fig.S6. Incidence and disability-adjusted life years (DALYs) rates of pertussis across countries with different SDI levels from 1990 to 2021.


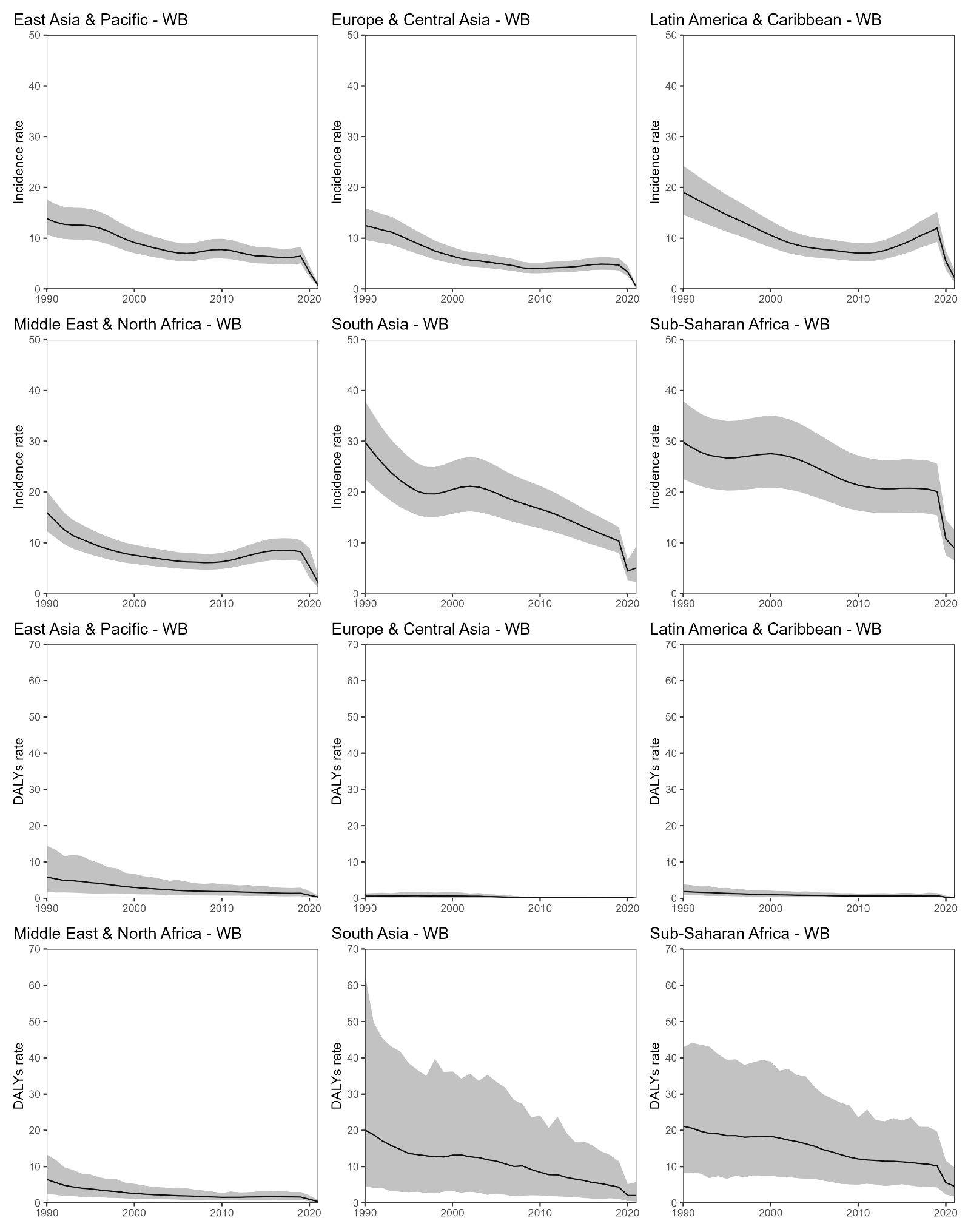


Fig.S7. Incidence and disability-adjusted life years (DALYs) rates of pertussis across countries in different regions from 1990 to 2021.

Table S1. Global adjusted annual percentage change (AAPC) in the incidence and disability-adjusted life years (DALYs) of pertussis among adults aged over 20 years, from 1990 to 2021, by different time periods bases joinpoint regression model.

| **Year** | **Incidence number, AAPC (95%CI)** | ***P* value** | **DALYs number, AAPC (95%CI)** | ***P* value** |
| --- | --- | --- | --- | --- |
| 1990–2021 | -4.21 (-4.36, -4.07) | <0.001 | -4.53 (-5.07, -3.99) | <0.001 |
| 1990–1999 | -1.75 (-1.93, -1.56) | <0.001 | -1.26 (-1.61, -0.92) | <0.001 |
| 1999–2009 | -0.34 (-0.52, -0.16) | <0.001 | -1.68 (-1.93, -1.42) | <0.001 |
| 2009–2019 | 0.38 (0.26, 0.50) | <0.001 | -2.29 (-2.72, -1.87) | <0.001 |
| 2019–2021 | -44.56 (-45.67, -43.43) | <0.001 | -36.93 (-41.96, -31.46) | <0.001 |

Table S2. Number, rate, and adjusted annual percentage change (AAPC) of pertussis incidence among adults aged over 20 years at the global and regional levels from 1990 to 2021, including subgroup analyses by sex, age group, sociodemographic index, and World Bank region, with estimates based on the joinpoint regression model.

| **Group** | **1990 Incidence** | **1990 Incidence rate (per 100,000)** | **2019 Incidence** | **2019 Incidence rate (per 100,000)** | **2021 Incidence** | **2021 Incidence rate (per 100,000)** | **Incidence number AAPC (95%CI) 1990–2019** | **Incidence number AAPC (95%CI) 2019–2021** | **Incidence rate AAPC (95%CI) 1990–2019** | **Incidence rate AAPC (95%CI) 2019–2021** |
| --- | --- | --- | --- | --- | --- | --- | --- | --- | --- | --- |
| Global | 536,314 (410,879, 682,625) | 17.44 (13.36, 22.20) | 461,505 (356,657, 587,642) | 9.00 (6.96, 11.46) | 142,012 (94,990, 211,508) | 2.70 (1.81, 4.02) | -0.53 (-0.64, -0.43)*** | -44.56 (-45.67, -43.43)*** | -2.28 (-2.39, -2.16)*** | -45.05 (-46.34, -43.74)*** |
| Female | 262,500 (201,077, 334,121) | 16.97 (13.00, 21.60) | 225,689 (174,484, 287,347) | 8.72 (6.74, 11.10) | 70,040 (46,791, 103,811) | 2.64 (1.76, 3.91) | -0.53 (-0.63, -0.43)*** | -44.41 (-45.48, -43.32)*** | -2.29 (-2.41, -2.18)*** | -44.92 (-46.20, -43.61)*** |
| Male | 273,815 (209,802, 348,505) | 17.92 (13.73, 22.80) | 235,816 (182,167, 300,295) | 9.29 (7.18, 11.83) | 71,972 (48,164, 107,704) | 2.77 (1.85, 4.14) | -0.54 (-0.65, -0.43)*** | -44.71 (-45.86, -43.53)*** | -2.26 (-2.38, -2.15)*** | -45.20 (-46.50, -43.87)*** |
| 20**–**24 years | 92,644 (70,885, 117,926) | 18.83 (14.41, 23.96) | 66,517 (51,471, 84,625) | 11.18 (8.65, 14.22) | 22,918 (15,445, 33,718) | 3.84  (2.59, 5.65) | -1.14 (-1.21, -1.07)*** | -42.23 (-43.43, -41.01)*** | -1.70 (-1.86, -1.53)*** | -42.10 (-44.76, -39.33)*** |
| 25**–**29 years | 76,400 (58,481, 97,255) | 17.26 (13.21, 21.97) | 58,164 (45,001, 74,018) | 9.77  (7.56, 12.43) | 19,152 (12,815, 28,318) | 3.26  (2.18, 4.81) | -0.88 (-0.96, -0.80)*** | -43.68 (-45.05, -42.28)*** | -1.95 (-2.12, -1.78)*** | -42.63 (-44.17, -41.05)*** |
| 30**–**34 years | 67,475 (51,666, 85,898) | 17.51 (13.41, 22.29) | 56,455 (43,655, 71,873) | 9.42  (7.29, 12.00) | 17,849 (11,910, 26,509) | 2.95  (1.97, 4.39) | -0.61 (-0.70, -0.51)*** | -44.04 (-45.53, -42.51)*** | -2.13 (-2.21, -2.04)*** | -44.26 (-45.73, -42.76)*** |
| 35**–**39 years | 66,691 (51,112, 84,886) | 18.93 (14.51, 24.10) | 56,049 (43,336, 71,348) | 10.44 (8.07, 13.29) | 17,619 (11,713, 26,305) | 3.14  (2.09, 4.69) | -0.62 (-0.81, -0.43)*** | -44.13 (-45.75, -42.45)*** | -2.00 (-2.09, -1.91)*** | -45.51 (-46.43, -44.57)*** |
| 40**–**44 years | 68,960 (52,857, 87,771) | 24.07 (18.45, 30.64) | 63,500 (49,071, 80,848) | 12.98 (10.03, 16.53) | 19,271 (12,823, 28,840) | 3.85  (2.56, 5.77) | -0.31 (-0.50, -0.12)** | -44.93 (-46.28, -43.54)*** | -2.10 (-2.31, -1.89)*** | -45.65 (-46.68, -44.59)*** |
| 45**–**49 years | 49,170 (37,673, 62,580) | 21.18 (16.22, 26.95) | 49,801 (38,456, 63,442) | 10.57 (8.16, 13.46) | 14,407 (9,667, 21,572) | 3.04  (2.04, 4.56) | 0.03  (-0.23, 0.29) | -46.02 (-47.19, -44.83)*** | -2.35 (-2.48, -2.22)*** | -46.35 (-47.00, -45.69)*** |
| 50**–**54 years | 43,852 (33,625, 55,807) | 20.63 (15.82, 26.25) | 42,587 (32,946, 54,275) | 9.80  (7.58, 12.49) | 11,939 (7,962, 17,939) | 2.68  (1.79, 4.03) | -0.07  (-0.19, 0.05) | -47.03 (-47.56, -46.50)*** | -2.53 (-2.74, -2.33)*** | -47.56 (-48.42, -46.69)*** |
| 55+ years | 71,121 (54,580, 90,503) | 10.59 (8.13, 13.48) | 68,431 (52,973, 87,217) | 4.87  (3.77, 6.20) | 18,855 (12,528, 28,391) | 1.27  (0.84, 1.91) | -0.14  (-0.48, 0.19) | -47.18 (-48.56, -45.77)*** | -2.64 (-2.88, -2.40)*** | -48.65 (-49.76, -47.53)*** |
| High | 44,872 (34,721, 56,998) | 7.14 (5.53, 9.07) | 33,773 (25,871, 43,498) | 3.96 (3.04, 5.10) | 3,397 (2,139, 5,515) | 0.39 (0.25, 0.64) | -1.81 (-2.26, -1.35)*** | -59.11 (-69.58, -45.03)*** | -2.88 (-3.33, -2.42)*** | -59.06 (-69.62, -44.81)*** |
| High-middle | 86,412 (66,894, 109,891) | 12.46 (9.65, 15.85) | 43,618 (33,653, 56,173) | 4.39 (3.38, 5.65) | 6,218 (4,108, 8,795) | 0.62 (0.41, 0.88) | -2.35 (-2.63, -2.07)*** | -59.84 (-63.34, -56.02)*** | -3.60 (-3.86, -3.35)*** | -59.55 (-62.83, -55.97)*** |
| Middle | 163,426 (125,833, 207,549) | 17.05 (13.13, 21.66) | 160,615 (124,013, 204,288) | 9.67 (7.47, 12.30) | 36,727 (22,923, 56,692) | 2.16 (1.35, 3.34) | -0.05  (-0.39, 0.29) | -51.31 (-52.57, -50.02)*** | -1.88 (-2.36, -1.40)*** | -52.48 (-54.19, -50.71)*** |
| Low-middle | 163,955 (124,248, 208,546) | 28.74 (21.78, 36.56) | 129,743 (100,381, 164,790) | 11.65 (9.01, 14.80) | 50,506 (29,414, 81,037) | 4.37 (2.54, 7.01) | -0.87 (-1.08, -0.66)*** | -39.70 (-41.85, -37.47)*** | -3.14 (-3.36, -2.92)*** | -40.67 (-42.96, -38.29)*** |
| Low | 77,273 (58,420, 97,967) | 34.85 (26.35, 44.18) | 93,310 (71,565, 118,787) | 18.59 (14.26, 23.67) | 45,130 (32,674, 62,258) | 8.46 (6.13, 11.68) | 0.63 (0.36, 0.91)*** | -30.75 (-36.49, -24.50)*** | -2.17 (-2.42, -1.92)*** | -32.94 (-38.22, -27.21)*** |
| East Asia & Pacific | 156,908 (121,223, 199,431) | 13.85 (10.70, 17.60) | 112,796 (87,627, 144,489) | 6.46 (5.02, 8.28) | 11,261 (6,855, 17,158) | 0.64 (0.39, 0.97) | -1.27 (-1.81, -0.73)*** | -65.79 (-71.25, -59.30)*** | -2.69 (-3.15, -2.24)*** | -65.77 (-70.48, -60.32)*** |
| Europe & Central Asia | 74,047 (57,188, 94,119) | 12.50 (9.65, 15.88) | 33,204 (25,698, 42,682) | 4.71 (3.65, 6.06) | 2,669 (1,767, 3,783) | 0.38 (0.25, 0.54) | -2.31 (-2.66, -1.96)*** | -68.95 (-71.98, -65.59)*** | -2.91 (-3.25, -2.57)*** | -68.92 (-71.85, -65.68)*** |
| Latin America & Caribbean | 44,585 (34,195, 56,711) | 19.08 (14.63, 24.26) | 52,481 (40,634, 66,562) | 11.97 (9.27, 15.19) | 9,761 (5,299, 16,676) | 2.17 (1.18, 3.70) | 0.62 (0.48, 0.76)*** | -56.75 (-57.57, -55.90)*** | -1.54 (-1.68, -1.41)*** | -57.35 (-57.83, -56.86)*** |
| Middle East & North Africa | 19,421 (14,975, 24,599) | 15.97 (12.32, 20.23) | 23,816 (18,441, 30,416) | 8.26 (6.40, 10.55) | 6,316 (3,568, 11,164) | 2.11 (1.19, 3.73) | 1.24 (0.84, 1.64)*** | -49.57 (-53.44, -45.38)*** | -1.73 (-2.11, -1.35)*** | -50.43 (-54.16, -46.39)*** |
| South Asia | 168,568 (127,619, 214,391) | 29.82 (22.57, 37.92) | 118,326 (91,404, 150,159) | 10.33 (7.98, 13.11) | 60,486 (26,582, 111,269) | 5.07 (2.23, 9.33) | -1.88 (-3.29, -0.44)* | -26.20 (-35.21, -15.93)*** | -4.24 (-5.62, -2.83)*** | -27.59 (-36.43, -17.53)*** |
| Sub-Saharan Africa | 67,046 (50,805, 85,276) | 29.80 (22.58, 37.91) | 104,999 (80,481, 133,686) | 20.11 (15.41, 25.60) | 49,437 (35,899, 69,536) | 8.92 (6.48, 12.54) | 1.52 (1.18, 1.85)*** | -31.74 (-38.48, -24.26)*** | -1.38 (-1.70, -1.06)*** | -33.90 (-40.23, -26.89)*** |

**P* <0.05, ***P*<0.01, ****P*<0.001.

Table S3. Number, rate, and adjusted annual percentage change (AAPC) of pertussis disability-adjusted life years (DALYs) among adults aged over 20 years at the global and regional levels from 1990 to 2021, including subgroup analyses by sex, age group, sociodemographic index, and World Bank region, with estimates based on the joinpoint regression model.

| **Group** | **1990 DALYs** | **1990 DALYs rate (per 100,000)** | **2019 DALYs** | **2019 DALYs rate (per 100,000)** | **2021 DALYs** | **2021 DALYs rate (per 100,000)** | **DALYs number AAPC (95%CI) 1990–2019** | **DALYs number AAPC (95%CI) 2019–2021** | **DALYs rate AAPC (95%CI) 1990–2019** | **DALYs rate AAPC (95%CI) 2019–2021** |
| --- | --- | --- | --- | --- | --- | --- | --- | --- | --- | --- |
| Global | 243,260 (97,525, 570,203) | 7.91 (3.17, 18.54) | 136,357 (65,951, 250,707) | 2.66 (1.29, 4.89) | 57,690 (23,499, 119,149) | 1.10 (0.45, 2.27) | -1.76 (-2.02, -1.50)*** | -36.93 (-41.96, -31.46)*** | -3.73 (-3.98, -3.48)*** | -37.28 (-40.09, -34.35)*** |
| Female | 115,407 (46,589, 267,125) | 7.46 (3.01, 17.27) | 67,890 (33,250, 122,840) | 2.62 (1.28, 4.74) | 28,108 (10,776, 58,684) | 1.06 (0.41, 2.21) | -1.59 (-1.85, -1.33)*** | -37.72 (-42.66, -32.35)*** | -3.57 (-3.83, -3.31)*** | -38.03 (-40.90, -35.02)*** |
| Male | 127,852 (50,045, 306,400) | 8.37 (3.27, 20.05) | 68,467 (32,624, 126,565) | 2.70 (1.29, 4.99) | 29,582 (12,612, 60,662) | 1.14 (0.48, 2.33) | -2.16 (-2.36, -1.96)*** | -36.05 (-38.34, -33.68)*** | -3.88 (-4.12, -3.63)*** | -36.55 (-39.30, -33.68)*** |
| 20**–**24 years | 56,266 (22,645, 129,727) | 11.43 (4.60, 26.36) | 30,572 (14,487, 54,861) | 5.14  (2.44, 9.22) | 12,281 (4,729, 26,019) | 2.06  (0.79, 4.36) | -2.10 (-2.32, -1.89)*** | -38.59 (-40.98, -36.10)*** | -2.49 (-3.05, -1.92)*** | -38.85 (-44.19, -33.00)*** |
| 25**–**29 years | 40,701 (16,262, 93,135) | 9.20  (3.67, 21.04) | 22,750 (10,842, 41,166) | 3.82  (1.82, 6.91) | 9,545 (3,785, 19,856) | 1.62  (0.64, 3.37) | -1.86 (-1.98, -1.75)*** | -38.38 (-42.76, -33.66)*** | -3.13 (-3.44, -2.81)*** | -35.14 (-38.74, -31.34)*** |
| 30**–**34 years | 32,837 (13,172, 76,484) | 8.52  (3.42, 19.84) | 18,826 (9,014, 34,534) | 3.14  (1.50, 5.76) | 8,206 (3,391, 17,076) | 1.36  (0.56, 2.82) | -1.73 (-1.95, -1.50)*** | -35.65 (-40.17, -30.79)*** | -3.34 (-3.55, -3.13)*** | -36.80 (-39.28, -34.22)*** |
| 35**–**39 years | 30,309 (11,952, 72,382) | 8.60  (3.39, 20.55) | 16,813 (8,099, 30,721) | 3.13  (1.51, 5.72) | 7,252 (2,988, 14,888) | 1.29  (0.53, 2.65) | -2.05 (-2.27, -1.84)*** | -35.91 (-38.41, -33.30)*** | -3.10 (-3.23, -2.98)*** | -38.69 (-43.59, -33.37)*** |
| 40**–**44 years | 29,234 (11,434, 69,707) | 10.20 (3.99, 24.33) | 16,920 (8,225, 31,014) | 3.46  (1.68, 6.34) | 7,295 (3,022, 14,978) | 1.46  (0.60, 2.99) | -1.76 (-1.97, -1.56)*** | -36.21 (-40.00, -32.19)*** | -3.50 (-3.65, -3.34)*** | -36.36 (-42.45, -29.63)*** |
| 45**–**49 years | 19,146 (7,549, 46,516) | 8.25  (3.25, 20.03) | 11,326 (5,632, 20,513) | 2.40  (1.20, 4.35) | 4,833 (2,023, 9,864) | 1.02  (0.43, 2.08) | -1.77 (-2.01, -1.53)*** | -36.79 (-39.28, -34.19)*** | -4.22 (-4.59, -3.85)*** | -36.56 (-41.83, -30.82)*** |
| 50**–**54 years | 14,390 (5,693, 34,024) | 6.77  (2.68, 16.01) | 8,145 (4,078, 14,689) | 1.87  (0.94, 3.38) | 3,556 (1,522, 7,124) | 0.80  (0.34, 1.60) | -1.95 (-2.23, -1.67)*** | -35.35 (-38.59, -31.95)*** | -4.25 (-4.32, -4.17)*** | -36.04 (-39.18, -32.73)*** |
| 55+ years | 20,378 (8,114, 48,756) | 3.03  (1.21, 7.26) | 11,006 (5,477, 20,059) | 0.78  (0.39, 1.43) | 4,721 (2,042, 9,827) | 0.32  (0.14, 0.66) | -2.09 (-2.23, -1.95)*** | -36.79 (-39.02, -34.48)*** | -4.35 (-4.47, -4.23)*** | -38.93 (-43.68, -33.78)*** |
| High | 2,032 (800, 4,269) | 0.32 (0.13, 0.68) | 718 (394, 1,303) | 0.08 (0.05, 0.15) | 141 (50, 301) | 0.02 (0.01, 0.03) | -3.51 (-3.75, -3.26)*** | -53.00 (-56.56, -49.14)*** | -4.49 (-4.71, -4.27)*** | -53.22 (-56.47, -49.72)*** |
| High-middle | 14,357 (4,517, 36,014) | 2.07 (0.65, 5.19) | 3,251 (1,556, 6,529) | 0.33 (0.16, 0.66) | 1,348 (417, 3,674) | 0.13 (0.04, 0.37) | -4.92 (-5.57, -4.28)*** | -35.19 (-40.17, -29.79)*** | -6.09 (-6.72, -5.46)*** | -35.40 (-40.27, -30.13)*** |
| Middle | 69,167 (24,805, 180,960) | 7.22 (2.59, 18.88) | 30,925 (14,827, 60,273) | 1.86 (0.89, 3.63) | 10,718 (3,782, 24,062) | 0.63 (0.22, 1.42) | -2.78 (-2.97, -2.58)*** | -41.77 (-43.94, -39.52)*** | -4.61 (-4.85, -4.37)*** | -42.22 (-44.72, -39.61)*** |
| Low-middle | 98,922 (31,687, 264,121) | 17.34 (5.56, 46.31) | 47,265 (19,107, 105,532) | 4.24 (1.72, 9.48) | 20,806 (6,926, 52,107) | 1.80 (0.60, 4.50) | -2.45 (-2.72, -2.17)*** | -37.06 (-40.18, -33.78)*** | -4.68 (-4.95, -4.42)*** | -38.08 (-41.14, -34.87)*** |
| Low | 58,633 (22,382, 132,760) | 26.44 (10.09, 59.88) | 54,044 (22,984, 105,203) | 10.77 (4.58, 20.96) | 24,659 (9,549, 51,718) | 4.62 (1.79, 9.70) | -0.37 (-0.68, -0.06)* | -32.71 (-38.95, -25.84)*** | -3.14 (-3.43, -2.85)*** | -34.85 (-40.67, -28.47)*** |
| East Asia & Pacific | 66,095 (20,874, 163,885) | 5.83 (1.84, 14.47) | 24,159 (9,868, 50,710) | 1.38 (0.57, 2.90) | 5,994 (1,610, 14,009) | 0.34 (0.09, 0.79) | -3.47 (-3.73, -3.21)*** | -48.37 (-52.60, -43.75)*** | -4.86 (-5.07, -4.65)*** | -48.37 (-51.76, -44.75)*** |
| Europe & Central Asia | 3,546 (1,411, 7,878) | 0.60 (0.24, 1.33) | 1,087 (522, 2,078) | 0.15 (0.07, 0.29) | 161 (55, 386) | 0.02 (0.01, 0.05) | -3.66 (-4.39, -2.93)*** | -59.04 (-64.56, -52.66)*** | -4.24 (-4.96, -3.50)*** | -59.06 (-64.59, -52.67)*** |
| Latin America & Caribbean | 4,345 (2,081, 8,943) | 1.86 (0.89, 3.83) | 3,016 (1,358, 6,240) | 0.69 (0.31, 1.42) | 571 (212, 1,549) | 0.13 (0.05, 0.34) | -1.32 (-1.52, -1.12)*** | -56.47 (-58.17, -54.70)*** | -3.44 (-3.64, -3.25)*** | -56.98 (-58.69, -55.20)*** |
| Middle East & North Africa | 7,869 (3,038, 16,210) | 6.47 (2.50, 13.33) | 4,656 (2,399, 8,673) | 1.62 (0.83, 3.01) | 1,035 (406, 2,328) | 0.35 (0.14, 0.78) | -1.24 (-1.68, -0.80)*** | -52.69 (-56.71, -48.30)*** | -4.16 (-4.59, -3.73)*** | -53.47 (-57.46, -49.11)*** |
| South Asia | 113,637 (25,880, 356,502) | 20.10 (4.58, 63.06) | 49,845 (12,872, 131,783) | 4.35 (1.12, 11.51) | 24,442 (4,855, 68,458) | 2.05 (0.41, 5.74) | -3.42 (-4.53, -2.30)*** | -26.97 (-34.05, -19.12)*** | -5.75 (-6.82, -4.66)*** | -28.34 (-35.30, -20.64)*** |
| Sub-Saharan Africa | 47,549 (18,714, 96,461) | 21.14 (8.32, 42.88) | 53,295 (22,260, 102,861) | 10.21 (4.26, 19.70) | 25,446 (9,737, 53,958) | 4.59 (1.76, 9.73) | 0.49  (0.20, 0.79)** | -33.33 (-36.66, -29.83)*** | -2.55 (-2.89, -2.22)*** | -33.57 (-40.38, -25.99)*** |

**P* <0.05, ***P*<0.01, ****P*<0.001.

Table S4. Adjusted annual percentage change (AAPC) in the numbers and rates of pertussis incidence across all adult age groups from 1990 to 2021, by different time periods and age subgroups, with estimates based on the joinpoint regression model.

| **Measure** | **Age group** | **1990–1999** | **1999–2009** | **2009–2019** | **2019–2021** | **1990–2021** |
| --- | --- | --- | --- | --- | --- | --- |
| Number | 20**–**24 years | -2.39 (-2.59, -2.19)*** | -0.57 (-0.63, -0.52)*** | -0.57 (-0.63, -0.52)*** | -42.23 (-43.43, -41.01)*** | -4.51 (-4.65, -4.37)*** |
| Number | 25**–**29 years | -1.71 (-1.81, -1.61)*** | -1.02 (-1.10, -0.95)*** | 0.02 (-0.11, 0.15) | -43.68 (-45.05, -42.28)*** | -4.43 (-4.59, -4.27)*** |
| Number | 30**–**34 years | -1.15 (-1.23, -1.07)*** | -1.15 (-1.23, -1.07)*** | 0.44 (0.19, 0.69)** | -44.04 (-45.53, -42.51)*** | -4.22 (-4.40, -4.04)*** |
| Number | 35**–**39 years | -1.56 (-1.87, -1.25)*** | -0.44 (-0.54, -0.34)*** | 0.05 (-0.39, 0.50) | -44.13 (-45.75, -42.45)*** | -4.25 (-4.49, -4.00)*** |
| Number | 40**–**44 years | -1.22 (-1.80, -0.63)*** | 0.10 (0.03, 0.17)* | 0.10 (0.03, 0.17)* | -44.93 (-46.28, -43.54)*** | -4.05 (-4.28, -3.83)*** |
| Number | 45**–**49 years | -0.72 (-1.19, -0.26)** | -0.07 (-0.58, 0.43) | 0.81 (0.59, 1.03)*** | -46.02 (-47.19, -44.83)*** | -3.88 (-4.14, -3.61)*** |
| Number | 50**–**54 years | -2.38 (-2.49, -2.28)*** | 0.40 (0.10, 0.69)** | 1.58 (1.44, 1.72)*** | -47.03 (-47.56, -46.50)*** | -4.08 (-4.21, -3.96)*** |
| Number | 55+ years | -2.70 (-2.90, -2.50)*** | 0.39 (-0.05, 0.84) | 1.66 (0.82, 2.51)*** | -47.18 (-48.56, -45.77)*** | -4.16 (-4.50, -3.83)*** |
| Rate | 20**–**24 years | -2.35 (-2.48, -2.21)*** | -2.35 (-2.48, -2.21)*** | -0.45 (-0.87, -0.02)* | -42.10 (-44.76, -39.33)*** | -5.00 (-5.31, -4.69)*** |
| Rate | 25**–**29 years | -3.00 (-3.28, -2.72)*** | -1.86 (-1.95, -1.76)*** | -1.10 (-1.50, -0.69)*** | -42.63 (-44.17, -41.05)*** | -5.29 (-5.50, -5.07)*** |
| Rate | 30**–**34 years | -3.66 (-3.89, -3.42)*** | -1.43 (-1.51, -1.36)*** | -1.43 (-1.51, -1.36)*** | -44.26 (-45.73, -42.76)*** | -5.62 (-5.79, -5.45)*** |
| Rate | 35**–**39 years | -3.43 (-3.58, -3.28)*** | -1.92 (-2.11, -1.73)*** | -0.78 (-0.88, -0.67)*** | -45.51 (-46.43, -44.57)*** | -5.64 (-5.77, -5.51)*** |
| Rate | 40**–**44 years | -3.63 (-3.82, -3.44)*** | -2.28 (-2.72, -1.84)*** | -0.53 (-0.83, -0.22)** | -45.65 (-46.68, -44.59)*** | -5.75 (-5.97, -5.53)*** |
| Rate | 45**–**49 years | -4.13 (-4.23, -4.02)*** | -2.20 (-2.39, -2.00)*** | -0.87 (-1.16, -0.58)*** | -46.35 (-47.00, -45.69)*** | -6.05 (-6.19, -5.91)*** |
| Rate | 50**–**54 years | -4.15 (-4.27, -4.03)*** | -2.60 (-2.84, -2.36)*** | -0.98 (-1.50, -0.46)*** | -47.56 (-48.42, -46.69)*** | -6.35 (-6.56, -6.14)*** |
| Rate | 55+ years | -4.54 (-4.70, -4.37)*** | -2.17 (-2.56, -1.78)*** | -1.37 (-1.88, -0.85)*** | -48.65 (-49.76, -47.53)*** | -6.57 (-6.82, -6.33)*** |

**P* <0.05, ***P*<0.01, ****P*<0.001.

Table S5. Adjusted annual percentage change (AAPC) in the numbers and rates of disability-adjusted life years (DALYs) across all adult age groups from 1990 to 2021, by different time periods and age subgroups, with estimates based on the joinpoint regression model.

| **Measure** | **Age group** | **1990–1999** | **1999–2009** | **2009–2019** | **2019–2021** | **1990–2021** |
| --- | --- | --- | --- | --- | --- | --- |
| Number | 20**–**24 years | -2.38 (-2.88, -1.87)*** | -1.52 (-1.74, -1.30)*** | -2.44 (-2.61, -2.28)*** | -38.59 (-40.98, -36.10)*** | -5.01 (-5.31, -4.71)*** |
| Number | 25**–**29 years | -1.86 (-1.98, -1.75)*** | -1.86 (-1.98, -1.75)*** | -1.86 (-1.98, -1.75)*** | -38.38 (-42.76, -33.66)*** | -4.76 (-5.21, -4.32)*** |
| Number | 30**–**34 years | -0.81 (-1.19, -0.43)*** | -1.81 (-2.03, -1.58)*** | -2.47 (-2.77, -2.17)*** | -35.65 (-40.17, -30.79)*** | -4.38 (-4.85, -3.90)*** |
| Number | 35**–**39 years | -2.12 (-2.62, -1.61)*** | -1.36 (-1.58, -1.14)*** | -2.68 (-2.85, -2.52)*** | -35.91 (-38.41, -33.30)*** | -4.70 (-5.00, -4.39)*** |
| Number | 40**–**44 years | -1.18 (-1.37, -0.98)*** | -1.33 (-1.50, -1.16)*** | -2.71 (-3.18, -2.25)*** | -36.21 (-40.00, -32.19)*** | -4.46 (-4.87, -4.06)*** |
| Number | 45**–**49 years | -1.85 (-2.44, -1.26)*** | -1.46 (-1.65, -1.26)*** | -2.02 (-2.15, -1.88)*** | -36.79 (-39.28, -34.19)*** | -4.53 (-4.85, -4.21)*** |
| Number | 50**–**54 years | -2.94 (-3.53, -2.35)*** | -1.11 (-1.46, -0.77)*** | -1.89 (-2.10, -1.68)*** | -35.35 (-38.59, -31.95)*** | -4.55 (-4.94, -4.16)*** |
| Number | 55+ years | -3.09 (-3.50, -2.68)*** | -1.64 (-1.72, -1.56)*** | -1.64 (-1.72, -1.56)*** | -36.79 (-39.02, -34.48)*** | -4.82 (-5.06, -4.57)*** |
| Rate | 20**–**24 years | -1.94 (-2.36, -1.52)*** | -3.41 (-4.73, -2.07)*** | -2.05 (-2.87, -1.23)*** | -38.85 (-44.19, -33.00)*** | -5.38 (-6.11, -4.64)*** |
| Rate | 25**–**29 years | -3.07 (-3.82, -2.31)*** | -2.73 (-3.02, -2.42)*** | -3.59 (-3.80, -3.38)*** | -35.14 (-38.74, -31.34)*** | -5.61 (-6.04, -5.17)*** |
| Rate | 30**–**34 years | -3.68 (-4.16, -3.19)*** | -2.60 (-2.81, -2.38)*** | -3.78 (-3.96, -3.60)*** | -36.80 (-39.28, -34.22)*** | -5.96 (-6.25, -5.66)*** |
| Rate | 35**–**39 years | -3.10 (-3.23, -2.98)*** | -3.10 (-3.23, -2.98)*** | -3.10 (-3.23, -2.98)*** | -38.69 (-43.59, -33.37)*** | -5.92 (-6.42, -5.42)*** |
| Rate | 40**–**44 years | -3.50 (-3.65, -3.34)*** | -3.50 (-3.65, -3.34)*** | -3.50 (-3.65, -3.34)*** | -36.36 (-42.45, -29.63)*** | -6.05 (-6.65, -5.46)*** |
| Rate | 45**–**49 years | -5.29 (-6.38, -4.19)*** | -3.73 (-3.90, -3.56)*** | -3.73 (-3.90, -3.56)*** | -36.56 (-41.83, -30.82)*** | -6.73 (-7.33, -6.13)*** |
| Rate | 50**–**54 years | -4.25 (-4.32, -4.17)*** | -4.25 (-4.32, -4.17)*** | -4.25 (-4.32, -4.17)*** | -36.04 (-39.18, -32.73)*** | -6.71 (-7.00, -6.41)*** |
| Rate | 55+ years | -4.35 (-4.47, -4.23)*** | -4.35 (-4.47, -4.23)*** | -4.35 (-4.47, -4.23)*** | -38.93 (-43.68, -33.78)*** | -7.08 (-7.56, -6.61)*** |

**P* <0.05, ***P*<0.01, ****P*<0.001.

Table S6. Adjusted annual percentage change (AAPC) in the numbers and rates of incidence across all sociodemographic index groups and World Bank region groups from 1990 to 2021, by different time periods and age subgroups, with estimates based on the joinpoint regression model.

| **Measure** | **Region** | **1990–1999** | **1999–2009** | **2009–2019** | **2019–2021** | **1990–2021** |
| --- | --- | --- | --- | --- | --- | --- |
| Number | High SDI | -1.81 (-2.26, -1.35)*** | -1.81 (-2.26, -1.35)*** | -1.81 (-2.26, -1.35)*** | -59.11 (-69.58, -45.03)*** | -7.20 (-8.92, -5.45)*** |
| Number | High-middle SDI | -5.33 (-5.73, -4.92)*** | -2.45 (-2.73, -2.17)*** | 0.51  (0.08, 0.94)* | -59.84 (-63.34, -56.02)*** | -7.79 (-8.36, -7.22)*** |
| Number | Middle SDI | -2.11 (-2.58, -1.63)*** | 0.93  (0.13, 1.74)* | 0.84  (0.60, 1.08)*** | -51.31 (-52.57, -50.02)*** | -4.58 (-4.92, -4.25)*** |
| Number | Low-middle SDI | -1.75 (-2.21, -1.30)*** | 0.04  (-0.21, 0.28) | -0.96 (-1.10, -0.83)*** | -39.70 (-41.85, -37.47)*** | -4.00 (-4.28, -3.71)*** |
| Number | Low SDI | 1.40  (0.88, 1.92)*** | 0.48  (0.22, 0.75)*** | 0.09  (-0.24, 0.43) | -30.75 (-36.49, -24.50)*** | -1.76 (-2.34, -1.18)*** |
| Number | East Asia & Pacific - WB | -2.98 (-3.77, -2.19)*** | -1.33 (-1.87, -0.79)*** | 0.35  (-0.46, 1.17) | -65.79 (-71.25, -59.30)*** | -7.80 (-8.88, -6.70)*** |
| Number | Europe & Central Asia - WB | -5.56 (-5.85, -5.28)*** | -5.56 (-5.85, -5.28)*** | 4.18  (3.21, 5.17)*** | -68.95 (-71.98, -65.59)*** | -9.27 (-9.92, -8.62)*** |
| Number | Latin America & Caribbean - WB | -3.30 (-3.51, -3.09)*** | -2.08 (-2.25, -1.92)*** | 7.16  (6.92, 7.40)*** | -56.75 (-57.57, -55.90)*** | -4.71 (-4.88, -4.55)*** |
| Number | Middle East & North Africa - WB | -4.18 (-4.94, -3.41)*** | 1.24  (0.67, 1.81)*** | 6.38  (5.81, 6.95)*** | -49.57 (-53.44, -45.38)*** | -3.21 (-3.80, -2.62)*** |
| Number | South Asia - WB | -1.80  (-4.68, 1.16) | 0.81  (-0.95, 2.60) | -4.56 (-6.15, -2.93)*** | -26.20 (-35.21, -15.93)*** | -3.66 (-5.35, -1.95)*** |
| Number | Sub-Saharan Africa - WB | 2.31  (1.60, 3.03)*** | 1.29  (0.98, 1.60)*** | 1.03  (0.67, 1.40)*** | -31.74 (-38.48, -24.26)*** | -1.05  (-1.75, -0.35)** |
| Rate | High SDI | -2.88 (-3.33, -2.42)*** | -2.88 (-3.33, -2.42)*** | -2.88 (-3.33, -2.42)*** | -59.06 (-69.62, -44.81)*** | -8.14 (-9.86, -6.39)*** |
| Rate | High-middle SDI | -6.75 (-7.12, -6.38)*** | -3.71 (-3.97, -3.45)*** | -0.57 (-0.96, -0.17)** | -59.55 (-62.83, -55.97)*** | -8.86 (-9.38, -8.33)*** |
| Rate | Middle SDI | -4.13 (-4.37, -3.90)*** | -1.32 (-2.04, -0.59)*** | -0.37  (-1.48, 0.75) | -52.48 (-54.19, -50.71)*** | -6.36 (-6.84, -5.89)*** |
| Rate | Low-middle SDI | -4.00 (-4.49, -3.52)*** | -2.27 (-2.53, -2.00)*** | -3.23 (-3.37, -3.08)*** | -40.67 (-42.96, -38.29)*** | -6.16 (-6.46, -5.85)*** |
| Rate | Low SDI | -1.25 (-1.73, -0.77)*** | -2.35 (-2.59, -2.10)*** | -2.81 (-3.12, -2.51)*** | -32.94 (-38.22, -27.21)*** | -4.52 (-5.05, -3.99)*** |
| Rate | East Asia & Pacific - WB | -4.41 (-5.01, -3.81)*** | -2.99 (-3.44, -2.54)*** | -0.82  (-1.59, -0.05)* | -65.77 (-70.48, -60.32)*** | -9.04 (-9.95, -8.12)*** |
| Rate | Europe & Central Asia - WB | -6.22 (-6.50, -5.94)*** | -6.22 (-6.50, -5.94)*** | 3.70  (2.77, 4.65)*** | -68.92 (-71.85, -65.68)*** | -9.79 (-10.41, -9.16)*** |
| Rate | Latin America & Caribbean - WB | -5.65 (-5.76, -5.54)*** | -4.46 (-4.60, -4.33)*** | 5.43 (5.07, 5.79)*** | -57.35 (-57.83, -56.86)*** | -6.72 (-6.85, -6.58)*** |
| Rate | Middle East & North Africa - WB | -7.18 (-7.89, -6.45)*** | -2.40 (-2.91, -1.89)*** | 4.15  (3.52, 4.79)*** | -50.43 (-54.16, -46.39)*** | -5.98 (-6.54, -5.41)*** |
| Rate | South Asia - WB | -4.13 (-6.94, -1.24)** | -1.68  (-3.40, 0.06) | -6.82 (-8.38, -5.24)*** | -27.59 (-36.43, -17.53)*** | -5.95 (-7.59, -4.28)*** |
| Rate | Sub-Saharan Africa - WB | -0.58  (-1.25, 0.10) | -1.61 (-1.90, -1.32)*** | -1.87 (-2.21, -1.52)*** | -33.90 (-40.23, -26.89)*** | -3.89 (-4.55, -3.23)*** |

**P* <0.05, ***P*<0.01, ****P*<0.001.

Table.S7. Adjusted annual percentage change (AAPC) in the numbers and rates of disability-adjusted life years (DALYs) across all sociodemographic index groups and World Bank region groups from 1990 to 2021, by different time periods and age subgroups, with estimates based on the joinpoint regression model.

| **Measure** | **Region** | **1990–1999** | **1999–2009** | **2009–2019** | **2019–2021** | **1990–2021** |
| --- | --- | --- | --- | --- | --- | --- |
| Number | High SDI | -5.53 (-5.84, -5.22)*** | -3.86  (-4.09, -3.62)*** | -1.29 (-1.70, -0.88)*** | -53.00 (-56.56, -49.14)*** | -7.88 (-8.37, -7.39)*** |
| Number | High-middle SDI | -4.21 (-5.13, -3.28)*** | -6.47  (-6.92, -6.03)*** | -4.00 (-5.50, -2.48)*** | -35.19 (-40.17, -29.79)*** | -7.25 (-7.98, -6.50)*** |
| Number | Middle SDI | -3.78 (-4.11, -3.45)*** | -1.39  (-1.61, -1.17)*** | -3.25 (-3.66, -2.83)*** | -41.77 (-43.94, -39.52)*** | -5.94 (-6.22, -5.66)*** |
| Number | Low-middle SDI | -2.73 (-3.36, -2.08)*** | -1.45  (-1.73, -1.17)*** | -3.19 (-3.39, -2.98)*** | -37.06 (-40.18, -33.78)*** | -5.17 (-5.55, -4.78)*** |
| Number | Low SDI | 0.98  (0.32, 1.64)** | -0.76  (-1.04, -0.47)*** | -1.18 (-1.52, -0.85)*** | -32.71 (-38.95, -25.84)*** | -2.86 (-3.50, -2.21)*** |
| Number | East Asia & Pacific - WB | -4.92 (-5.27, -4.58)*** | -3.72  (-3.98, -3.46)*** | -1.88 (-2.32, -1.44)*** | -48.37 (-52.60, -43.75)*** | -7.29 (-7.83, -6.75)*** |
| Number | Europe & Central Asia - WB | 0.70  (-0.16, 1.57) | -10.50 (-11.45, -9.54)*** | -0.35  (-1.82, 1.14) | -59.04 (-64.56, -52.66)*** | -8.83 (-9.86, -7.80)*** |
| Number | Latin America & Caribbean - WB | -3.93 (-4.23, -3.63)*** | -2.39  (-2.69, -2.09)*** | 2.20  (1.82, 2.58)*** | -56.47 (-58.17, -54.70)*** | -6.40 (-6.68, -6.11)*** |
| Number | Middle East & North Africa - WB | -5.20 (-5.85, -4.54)*** | -1.83  (-2.51, -1.15)*** | 3.09  (2.24, 3.94)*** | -52.69 (-56.71, -48.30)*** | -5.82 (-6.46, -5.17)*** |
| Number | South Asia - WB | -2.49  (-4.96, 0.05) | -1.14  (-2.28, 0.02) | -6.47 (-7.70, -5.22)*** | -26.97 (-34.05, -19.12)*** | -5.15 (-6.46, -3.82)*** |
| Number | Sub-Saharan Africa - WB | 1.54  (1.27, 1.82)*** | -0.71  (-1.36, -0.06)* | 0.77  (0.30, 1.24)** | -33.33 (-36.66, -29.83)*** | -2.13 (-2.54, -1.73)*** |
| Rate | High SDI | -6.38 (-6.63, -6.12)*** | -5.12  (-5.33, -4.91)*** | -2.12 (-2.54, -1.71)*** | -53.22 (-56.47, -49.72)*** | -8.79 (-9.24, -8.34)*** |
| Rate | High-middle SDI | -5.66 (-6.54, -4.77)*** | -7.82  (-8.22, -7.41)*** | -4.74 (-6.23, -3.23)*** | -35.40 (-40.27, -30.13)*** | -8.33 (-9.05, -7.61)*** |
| Rate | Middle SDI | -5.65 (-6.03, -5.27)*** | -3.49  (-3.69, -3.29)*** | -4.78 (-5.31, -4.24)*** | -42.22 (-44.72, -39.61)*** | -7.64 (-7.97, -7.31)*** |
| Rate | Low-middle SDI | -4.96 (-5.58, -4.33)*** | -3.71  (-3.98, -3.44)*** | -5.40 (-5.60, -5.19)*** | -38.08 (-41.14, -34.87)*** | -7.30 (-7.67, -6.92)*** |
| Rate | Low SDI | -1.64 (-2.26, -1.02)*** | -3.57  (-3.83, -3.30)*** | -4.04 (-4.36, -3.73)*** | -34.85 (-40.67, -28.47)*** | -5.59 (-6.19, -4.98)*** |
| Rate | East Asia & Pacific - WB | -6.34 (-6.59, -6.10)*** | -5.35  (-5.55, -5.16)*** | -2.99 (-3.38, -2.60)*** | -48.37 (-51.76, -44.75)*** | -8.54 (-8.96, -8.11)*** |
| Rate | Europe & Central Asia - WB | 0.05  (-0.81, 0.91) | -11.13 (-12.08, -10.18)*** | -0.79  (-2.26, 0.70) | -59.06 (-64.59, -52.67)*** | -9.35 (-10.37, -8.31)*** |
| Rate | Latin America & Caribbean - WB | -6.27 (-6.57, -5.97)*** | -4.53  (-4.83, -4.23)*** | 0.30  (-0.08, 0.68) | -56.98 (-58.69, -55.20)*** | -8.35 (-8.64, -8.06)*** |
| Rate | Middle East & North Africa - WB | -8.37 (-9.12, -7.62)*** | -5.08  (-5.74, -4.42)*** | 0.76  (-0.08, 1.60) | -53.47 (-57.46, -49.11)*** | -8.53 (-9.15, -7.90)*** |
| Rate | South Asia - WB | -4.80 (-7.22, -2.33)*** | -3.58  (-4.69, -2.44)*** | -8.69 (-9.89, -7.47)*** | -28.34 (-35.30, -20.64)*** | -7.40 (-8.68, -6.10)*** |
| Rate | Sub-Saharan Africa - WB | -1.47 (-2.18, -0.74)*** | -2.86  (-3.17, -2.55)*** | -3.21 (-3.58, -2.84)*** | -33.57 (-40.38, -25.99)*** | -4.93 (-5.63, -4.23)*** |

**P* <0.05, ***P*<0.01, ****P*<0.001.
